# The application of chiroptical spectroscopy (circular dichroism) in quantifying binding events in lanthanide directed synthesis of chiral luminescent self-assembly structures†
†Electronic supplementary information (ESI) available. CCDC 999267–999270 and 1026036. For ESI and crystallographic data in CIF or other electronic format see DOI: 10.1039/c4sc02474e



**DOI:** 10.1039/c4sc02474e

**Published:** 2014-10-10

**Authors:** Oxana Kotova, Salvador Blasco, Brendan Twamley, John O'Brien, Robert D. Peacock, Jonathan A. Kitchen, Miguel Martínez-Calvo, Thorfinnur Gunnlaugsson

**Affiliations:** a School of Chemistry , Trinity Biomedical Sciences Institute , Trinity College Dublin , Dublin 2 , Ireland . Email: kotovao@tcd.ie ; Email: gunnlaut@tcd.ie; b School of Chemistry , University of Glasgow , Glasgow , G12 8QQ , Scotland , UK; c Chemistry , University of Southampton , Southampton , SO17 1BJ , UK

## Abstract

The binding of asymmetrical and optically pure tridentate ligands (**L** = **1(*S*)** and **1(*R*)**) containing one carboxylic group and 2-naphthyl as an antenna to lanthanide ions (**M** = La(iii) and Eu(iii)) was studied in CH_3_CN, showing the successive formation of **M:L**, **M:L_2_** and **M:L_3_** stoichiometric species in solution. The europium complexes **EuL_3_** were also synthesised, structurally characterised and their photophysical properties probed in CH_3_OH and CH_3_CN. The changes in the chiroptical properties of both **1(*S*)** and **1(*R*)** were used (by circular dichroism (CD) spectroscopy) to monitor the formation of these chiral self-assemblies in solution. While circularly polarised luminescence (CPL) showed the formation of **Eu(1(*S*))_3_** and **Eu(1(*R*))_3_** as enantiomers, with high luminescence dissymmetry factors (*g*_lum_), fitting the CD changes allowed for binding constants to be determined that were comparable to those seen in the analyses of absorbance and luminescence changes.

## Abstract 2

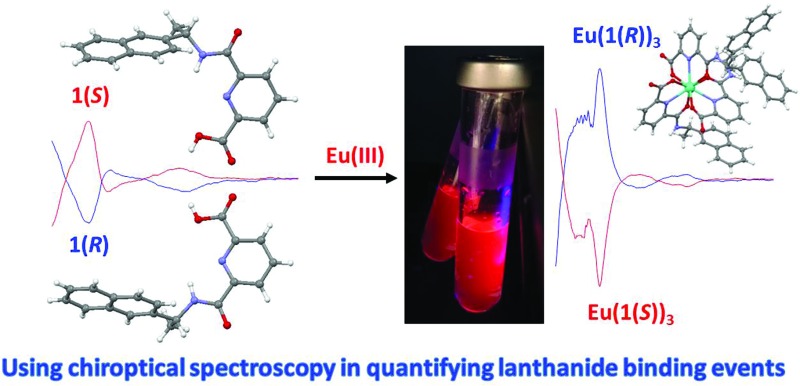
The binding of asymmetrical and optically pure tridentate ligands containing one carboxylic group and 2-naphthyl as an antenna to lanthanide ions was studied in CH_3_CN.

## Introduction

**Scheme 1:**
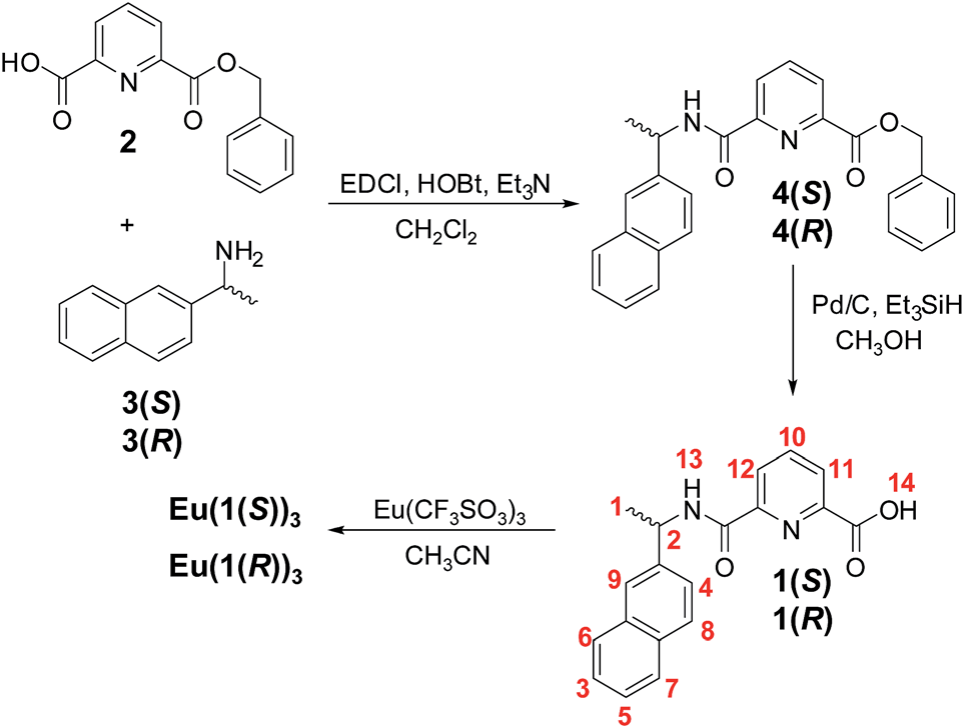
Synthesis of the ligands **1(*S*)** and **1(*R*)**, and their corresponding complexes **Eu(1(*S*))_3_** and **Eu(1(*R*))_3_** synthesized under microwave irradiation.

Due to the many unique spectroscopic properties that the lanthanides (Ln) possess, such as long-lived excited states and long emission wavelength, the synthesis of novel luminescent lanthanide based self-assemblies has led to the development of various novel optical and functional materials in recent times.^1^ Examples of such developments are the formation of supramolecular self-assembly structures, such as helicates and interlocked lanthanide-based catenanes,^1e,f^ luminescent sensors for ions and molecules, probes for cellular imaging and for observing biological process and as supramolecular polymers.^2^ Chiral lanthanide complexes have been developed increasingly for such applications.^3,4^ We have developed numerous examples of chiral ligands that have been employed in lanthanide directed synthesis of self-assembled architectures. These have been based on the dipicolinic acid motive (**H_2_dpa**)^5^ which has been shown to be an efficient sensitiser for Eu(iii) and Tb(iii) luminescence.^6^ Moreover, as we and others have demonstrated, the carboxylic groups can be synthetically modified easily with various chiral or achiral antennae to give cationic lanthanide complexes.^7–12^ In related work, both Bünzli and De Cola have recently demonstrated that mono-anionic asymmetrical ligands containing three donor atoms can form stable charge neutral complexes with various lanthanides.^13^ Herein we present ligands **1(*S*)** and **1(*R*)** (Scheme 1) based on the **H_2_dpa** core, each ligand possessing a single chiral (*S*)- and (*R*)-1-(2-naphthyl)-ethylamine antenna, respectively. Both Eu(iii) complexes were formed from these ligands using a synthesis under microwave irradiation and the photophysical properties of the resulting complexes **Eu(1(*S*))_3_** and **Eu(1(*R*))_3_** studied in CH_3_CN and CH_3_OH solutions. Self-assembly of these ligands with Ln(III) was studied in CH_3_CN solution by monitoring the changes in both ground and excited states (Ln = Eu(iii)) as well as by NMR spectroscopy (Ln = La(iii)). Due to the chiral nature of the ligands and concomitant formation of chiral Ln-complexes in solution upon titration with Ln(iii), we studied their chiroptical properties using both CD and CPL spectroscopies. The crystallographic analysis of **Eu(1(*R*))_3_** confirmed that the chirality of the ligand was transferred to the Eu(iii) centre. Adequately, the changes in CD spectra of either **1(*S*)** and **1(*R*)** were drastically affected upon binding to Eu(iii); being equal magnitude but of opposite signs. This allowed us to identify both the different **Ln_x_:L_y_** stoichiometries in solution and to quantify their binding affinities using non-linear regression analysis, in a similar manner to that used to quantify the same in both the absorption and the luminescence titrations. While CD spectroscopy is commonly used to monitor the interaction, formation or folding of larger self-assembly structures in solution,^14^ to the best of our knowledge, carrying out CD titrations to assess the different equilibrium processes for the formation of chiral self-assembly metal ion complexes and determination of the affinity constants for such processes is relatively unexplored in supramolecular chemistry. Here we demonstrate that indeed such analysis gives greater understanding of the different stoichiometric speciation in solution and that the binding constants determined for these matched comfortably well with that determined by traditional spectroscopic methods.

## Results and discussion

### Synthesis of the ligands and Eu(iii) complexes

**Fig. 1:**
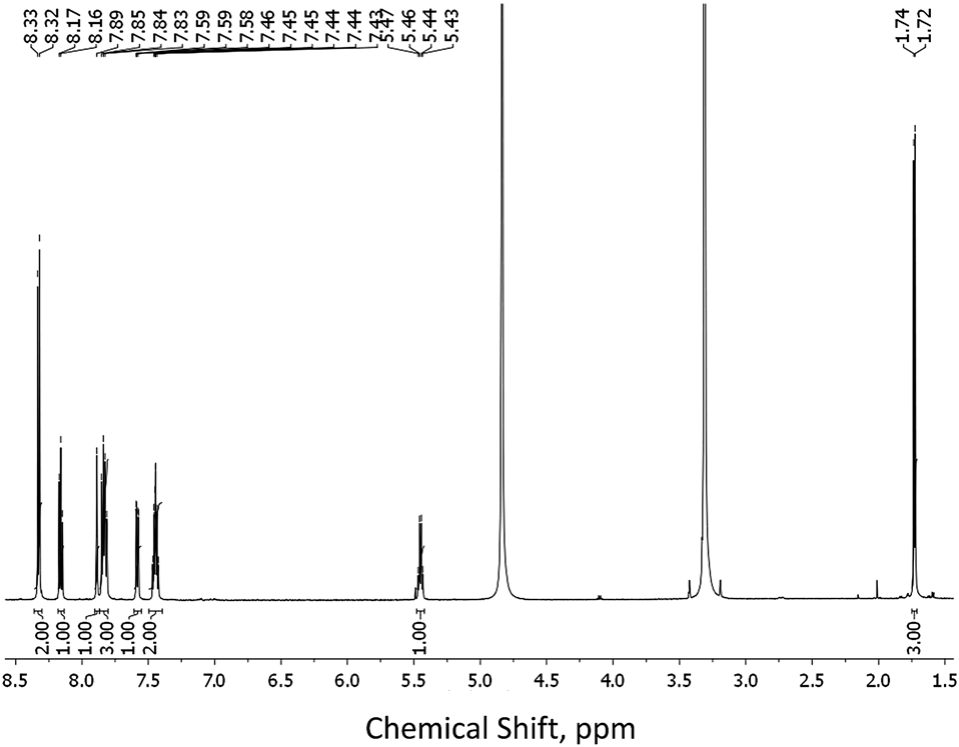
^1^H NMR of **1(*S*)** (600 MHz, CD_3_OD).

The tridentate ligands **1(*S*)** and **1(*R*)** were synthesised in three steps starting from commercially available **H_2_dpa**. First the dipicolinic acid was monoprotected with benzylbromide to give **2** in 38% yields (see ESI†).^15^ This was further coupled, Scheme 1, with either (*S*)- or (*R*)-1-(2-naphthyl)-ethylamine (**3(*S*)** or **3(*R*)**, respectively) using standard peptide-coupling methodology^9*b*^ in presence of 1-(3-dimethylaminopropyl)-3-ethylcarbodiimide hydrochloride (EDCI·HCl) and 1-hydroxybenzotriazole hydrate (HOBt) to give the intermediates **4(*S*)** and **4(*R*)** in *ca.* 80% yield for both. The deprotection of the benzyl esters **4(*S*)** and **4(*R*)** was first attempted by catalytic hydrogenation using 10% Pd/C catalyst. However, this resulted in the product containing impurities that were difficult to isolate from the desired products. For this reason we turned to the use of Pd–C-induced catalytic transfer hydrogenation using triethylsilane^16^ which gave desired products **1(*S*)** and **1(*R*)** in *ca.* 78% yield for both. The ^1^H NMR spectrum of **1(*S*)** is shown in Fig. 1, demonstrating the successful deprotection of the benzyl ester (the same result observed for **1(*R*)**; see ESI†). High resolution electrospray mass-spectrometry (see ESI†) also confirmed the successful formation of both ligands with of *m*/*z* = 319.1075 for **1(*S*)** and 319.1080 for **1(*R*)** corresponding to [M – H^+^]^–^, while the IR spectra revealed the presence of characteristic C–H, N–H and C=O vibrations. The results of the elemental analysis also confirmed the formation of the pure compounds. We were also able to grow single crystals suitable for X-ray crystal structure analysis of both **4(*S*)** and **4(*R*)** by recrystallization of powder samples from methanol. Similarly, single crystals of **1(*S*)** and **1(*R*)** were grown from a mixture of CH_3_OH and CH_3_CN and the low temperature (100(2) K) X-ray structures collected. These will be discussed in the next section.

Having obtained both **1(*S*)** and **1(*R*)** we next synthesised the Eu(iii) complexes **Eu(1(*S*))_3_** and **Eu(1(*R*))_3_** from these, where each of the ligand is expected to coordinate to the lanthanide *via* the central pyridine nitrogen, the carboxylic acid and the carboxylic amide, in a tri-dentate manner. Three of these ligands therefore fulfil the high coordination requirements of the lanthanide. This was achieved by reacting Eu(CF_3_SO_3_)_3_ with **1(*S*)** and **1(*R*)** in a 1 : 3 metal to ligand stoichiometry in CH_3_CN solution under microwave irradiation at 95 °C for 30 minutes. The resulting complexes were isolated as white powders by slow diffusion of diethyl ether into the CH_3_CN solutions, yielding the desired complexes in *ca.* 35% yields. High resolution matrix-assisted laser desorption/ionization (MALDI) mass-spectrometry confirmed the formation of both tris-chelates with the presence of *m*/*z* = 1147.2034 which was assigned to [Eu(**1(*S*)**–H^+^)_3_ + K^+^]^+^ and 1147.2039, assigned to [Eu(**1(*R*)**–H^+^)_3_ + K^+^]^+^ (see ESI†). The ^1^H NMR (CD_3_CN, 600 MHz) spectra for both Eu(iii) complexes were different to that of the free ligands showing significant broadening and shift of the proton resonances (see ESI†), due to the paramagnetic nature of the lanthanide ion. Similarly, in the IR spectra, the C=O vibronic transitions were significantly shifted by 126 cm^–1^ upon complexation to Eu(iii), further confirming the complex formation. Elemental analysis also confirmed the formation of the desired products. However, the latter, along with the MALDI results, suggests that partial deprotonation of the ligands upon complexation occurs, which is not surprising since the carboxylic acid is directly bound to the lanthanide, making it more acidic. Both complexes were also shown to be luminescent, as upon placing a solution of both under a UV light irradiation red emission characteristic of Eu(iii) was observed, confirming that the ligands functions as sensitising antennae for the ^5^D_0_ excited state of Eu(iii). Clear colourless crystals of **Eu(1(*R*))_3_** were obtained as described previously by Bünzli *et al.* ^13a–c^

The coordination geometry of the Eu(iii) centres was first evaluated in solution by measuring the decay of the lanthanide excited state in both H_2_O and D_2_O, upon excitation at the naphthalene antennae (*λ*_ex_ = 281 nm) allowing for the determination of the Eu(iii) hydration state (*q*, the number of metal-bound water molecules).^17^ The Eu(iii) ^5^D_0_ excited state life-times of **Eu(1(*S*))_3_** were best-fitted to monoexponential decay with *τ*_H_2_O_ = 1.54 ± 0.01 ms and *τ*_D_2_O_ = 2.57 ± 0.01 ms giving a *q* value of zero. This is to be expected as the lanthanides have coordination requirements of 8–10, these being fulfilled by the 9 coordination environment of the 1 : 3 **Eu:L** stoichiometry in **Eu(1(*S*))_3_**.^11^ Similarly, the Eu(iii) excited state life-times of **Eu(1(*R*))_3_** were best fitted to monoexponential decay with *τ*_H_2_O_ = 1.55 ± 0.01 ms and *τ*_D_2_O_ = 2.50 ± 0.04 ms, again confirming that the ions were complexed with saturation of coordination environment.

### Crystal and molecular structures of **4(*S*)**, **4(*R*)**, **1(*S*)**, **1(*R*)** and **Eu(1(*R*))_3_**

**Fig. 2:**
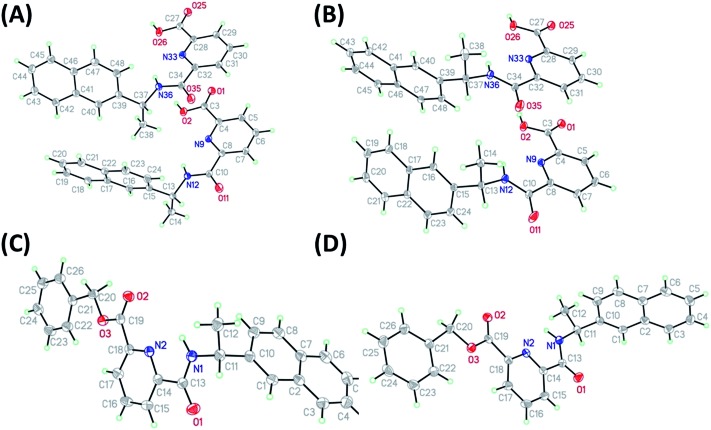
Molecular structures of (A) **1(*R*)** and (B) **1(*S*)** showing both independent molecules in the asymmmetric unit, (C) **4(*R*)** and (D) **4(*S*)** (thermal displacement 50%).

As stated above, crystalline materials of both **1(*R*)** and **1(*S*)** were obtained that allowed for the X-ray analysis of these enantiomers. The resulting structures are shown in Fig. 2A and B, respectively. Each enantiomer crystallizes in the chiral space group *P*2_1_ and displays two different independent molecules in the asymmetric unit. The chiral centres are C13 and C37, and although the chirality was known throughout the syntheses, it was confirmed by the refined Flack parameter for each enantiomer. The two independent molecules differ by rotation of the naphthyl group around the C13–C15 (C37–39) bond (torsion angle C14–C13–C15–C16, C38–C37–C39–C40; **1(*R*)** = –19.5(2), 9.3(2): **1(*S*)** = 19.84(17), –9.50(17)°). The structures of **4(*S*)** and **4(*R*)**, again in the chiral space group *P*2_1_, were obtained from enantiopure crystals grown from methanol solutions, see Fig. 2C and D, respectively. Here the asymmetric unit consists of a single molecule. The additional substitution on the carboxyl group leads to a planar pyridine–C(O)O–CH_2_–Ph unit (**4(*S*)**, **4(*R*)** = 0.04 Å deviation from plane). The chiral centre is C11 and was confirmed by the Flack parameter. The naphthyl groups are rotated about the C10–C11 bond with C9–C10–C11–C12 torsion angles of **4(*R*)**: –62.7(2) and **4(*S*)**: 62.8(2)°. The chiral subunit (R–C(O)NHCMe–naphthyl) seen in **1(*R*)**, **1(*S*)**, **4(*S*)** and **4(*R*)** has been structurally characterized previously and displays a wide range of naphthyl group: chiral centre arrangements.^18^

In both **1(*S*)** and **1(*R*)**, strong hydrogen bonding was observed between the carboxylic acid group and the carbonyl group of neighbouring ligand. This orientation results in other weak inter- and intra- molecular hydrogen bonding between the back-to-back molecules (see Table 1) and creates a weakly connected supramolecular ribbon motif parallel to the *c*-axis. In **4(*S*)** and **4(*R*)** the hydrogen bonding motif is disrupted by the substitution on the carboxyl group. In this case only weak C–H···O interactions prevail.

**Table 1:** Summary of crystallographic details for compounds **1(*S*)**, **1(*R*)**, **4(*S*)**, **4(*R*)** and **Eu(1(*R*))_3_**

Compound reference	**1(*R*)**	**1(*S*)**	**4(*R*)**	**4(*S*)**	**Eu(1(*R*))_3_**
Chemical formula	C_19_H_16_N_2_O_3_	C_19_H_16_N_2_O_3_	C_26_H_22_N_2_O_3_	C_26_H_22_N_2_O_3_	C_57_H_49.5_EuN_6_O_11.5_
Formula mass	320.34	320.34	410.45	410.45	1159.49
Crystal system	Monoclinic	Monoclinic	Monoclinic	Monoclinic	Orthorhombic
*a*/Å	6.8436(4)	6.8507(4)	5.4249(2)	5.4288(2)	26.0015(12)
*b*/Å	26.8023(14)	26.7770(17)	16.5948(6)	16.6067(6)	26.4029(11)
*c*/Å	8.3932(5)	8.4103(5)	11.4170(4)	11.4114(4)	30.8305(11)
*β*/°	90.9000(13)	90.840(2)	98.277(2)	98.2867(18)	90
Unit cell volume/Å^3^	1539.33(15)	1542.63(16)	1017.11(6)	1018.05(6)	21165.6(15)
Temperature/K	100(2)	100(2)	100(2)	100(2)	100(2)
Space group	*P*2_1_	*P*2_1_	*P*2_1_	*P*2_1_	*C*222_1_
No. of formula units per unit cell, *Z*	4	4	2	2	16
Radiation type	MoKα	MoKα	CuKα	CuKα	CuKα
Absorption coefficient, *μ*/mm^–1^	0.095	0.095	0.710	0.709	9.05
No. of reflections measured	24 241	47 256	5614	5359	36 491
No. of independent reflections	6995	10 624	2414	2462	9417
*R*_int_	0.0171	0.0145	0.0273	0.0303	0.0740
Final *R*_1_ values (*I* > 2*σ*(*I*))	0.0254	0.0321	0.0306	0.0309	0.083
Final w*R*(*F* ^2^) values (*I* > 2*σ*(*I*))	0.0646	0.0861	0.0806	0.0762	0.262
Final *R*_1_ values (all data)	0.0268	0.0339	0.0307	0.0310	0.1512
Final w*R*(*F* ^2^) values (all data)	0.0654	0.0873	0.0808	0.0763	0.2618
Goodness of fit on *F* ^2^	1.039	1.035	1.068	1.055	1.006
Flack parameter	0.0(2)	0.31(11)	–0.11(7)	–0.15(9)	–0.001(8)
CCDC number	999267	999268	999269	999270	1026036

**Fig. 3:**
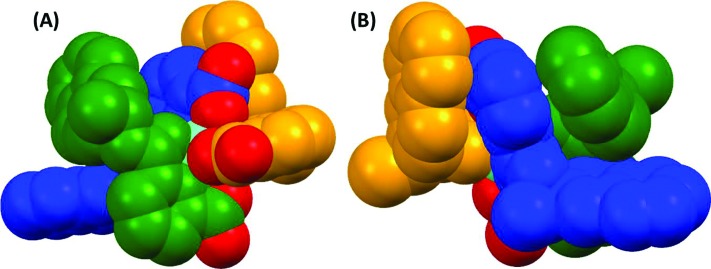
Space filling representation of **Eu(1(*R*))_3_** complex showing (A) the position of carboxyl oxygen atoms (in red) relatively to naphthyl antennae and (B) stacking interaction between pyridine and naphthyl groups.

The complex **Eu(1(*R*))_3_** (Fig. 3, ESI†) crystallized in an orthorhombic crystal system with chiral space group *C*222_1_ (Table 1). The asymmetric unit contains two different **Eu(1(*R*))_3_** molecules and some water molecules. The ligands are arranged around Eu(iii) ion in a manner predicted by us previously for the complexes formed from the use of ligands with 1-naphthyl group as an antenna (assigned here as **Eu(5(*S*))_3_** and **Eu(5(*R*))_3_**, see ESI† for structure)^11^ and observed by others^13^ with three naphthyl antennae located on the same side (Fig. 3A). As observed previously (*q* = 0) and confirmed here, the coordination environment of Eu(iii) centre in either of the molecules is fully saturated with three molecules of deprotonated **1(*R*)** contributing with three coordination bonds each being the pyridine nitrogen flanked by the carboxylate oxygen on one side and the amido oxygen on the other side. Both europium atoms are positioned in a nine-coordinated tri-capped trigonal prismatic N_3_O_6_ coordination environment formed by the three pyridine nitrogen atoms located in the equatorial plane arranging in quite regular triangle, and six oxygen atoms where three are placed above while the other three lay below the equatorial plane forming a triangular prism among them. Thus, the complex remains *C*_3_ symmetry. The average bond distances are 2.37 Å for Eu–O(carboxylate) bonds, 2.44 Å for Eu–O(amido) and 2.57 Å for Eu–N(pyridine). The chirality of the ligand, known from the synthesis, is also confirmed in this structure by the Flack parameter.

The naphthalene moieties interact with each other and with the pyridine groups *via* π-stacking and they form hydrophobic pockets in the structure. Multiple intramolecular and intermolecular π-stacking interactions can be observed in the fragment containing the Eu1 atom, the naphthalene C48–C57 interacts face to face with pyridine N3–C21–C25 and edge to face with naphthalene C29–C38 but this pyridine also interacts face to face with the naphthalene C10–C19 (Fig. 3B, ESI†). Similar interactions can be seen in the other fragment (the one that contains the Eu2 atom) with the pyridine N9–C78–C82 in between the naphthalene group C67–C76 and C116–C125. Naphthalene groups have some conformational freedom and they can, at some extent rock and rotate and as a result high disorder is found in these moieties. In the case of the naphthalene group attached to C(103) it has been found in two different orientations with occupancy factor 1/2.

### Photophysical properties of Eu(iii) complexes in CH_3_CN and CH_3_OH solutions

Having structurally characterised the ligands and their corresponding complexes, we next investigated their photophysical properties. The electronic absorption spectra of the ligands in CH_3_CN (see ESI†) were dominated by ligand-centred n → π* and π → π* transitions centred at 222 nm (**1(*S*)**: *ε* = 87 397 ± 230 cm^–1^ M^–1^; **1(*R*)**: *ε* = 87 158 ± 320 cm^–1^ M^–1^) and 270 nm (**1(*S*)**: *ε* = 10 964 ± 38 cm^–1^ M^–1^; **1(*R*)**: *ε* = 11 630 ± 146 cm^–1^ M^–1^). The excitation into the 270 nm transition did not result in any significant ligand centred emission. However, upon complexation to the metal ion significant Eu(iii)-centred emission was observed due to the energy transfer processes occurring from the 2-naphthyl antennae to the ^5^D_0_ excited state which was followed by deactivation to the ^7^F_*J*_ bands of the lanthanide. The photophysical properties of **Eu(1(*S*))_3_** and **Eu(1(*R*))_3_** were also studied in CH_3_OH and compared to that observed for **Eu(5(*S*))_3_** and **Eu(5(*R*))_3_** (ESI†).^11^ The results are shown in Table 2 and it was demonstrated that the structure of Eu(iii)-centred emission bands is similar for both, confirming that these systems have similar coordination environments of the metal centre. The photoluminescence quantum yields (*Φ*_tot_, %) were also measured by a relative method using Cs_3_[Eu(**dpa**)_3_]·9H_2_O as a standard.^19^ In general, the quantum yields of the 1-naphthyl derivatives were *ca.* 4 times higher in CH_3_CN and 2 times higher in CH_3_OH than seen for the 2-naphthyl analogues. Based on our previous work, where we investigated the symmetrical Eu(iii) “Trinity Sliotar” complexes,^8^ we believe that this difference is due to sensitisation efficiency of Eu(iii) luminescence being more favourable for **Eu(5(*S*))_3_** and **Eu(5(*R*))_3_** than **Eu(1(*S*))_3_** and **Eu(1(*R*))_3_**. This is evident from calculating the antenna-to-ion energy transfer efficiencies (*η*_sens_) for these complexes, which is determined on the basis of the emission spectrum, the observed luminescence life-time (*τ*_obs_) and the experimental overall luminescence quantum yield (*Φ*_tot_) upon ligand excitation, Table 2.

**Table 2:** Antenna-to-ion energy transfer efficiencies (*η*_sens_) of Eu(iii) complexes calculated on the basis of the observed emission spectrum, the observed luminescence life-time (*τ*_obs_) and the experimental overall luminescence quantum yield (*Φ*_tot_) upon ligand excitation (*λ*_ex_ = 279 nm). *τ*_R_ is the radiative life-time calculated using eqn (3) (see EP). *Φ*LnLn is found using eqn (4) (see EP) measured in CH_3_CN and CH_3_OH

Complex	*τ*_obs_, ms	*Φ*_tot_, %	*τ*_R_, ms	*Φ* Ln Ln , %	*η*_sens_, %	Solvent/*c*, M
**Eu(1(*R*))_3_**	1.86 ± 0.02	2.1 ± 0.1	6.43 ± 0.04	28.94 ± 0.01	7.26 ± 0.02	CH_3_CN 2.72 × 10^–5^ M
**Eu(1(*S*))_3_**	1.86 ± 0.02	1.6 ± 0.5	6.39 ± 0.04	29.13 ± 0.01	5.49 ± 0.02	
**Eu(5(*R*))_3_**	1.94 ± 0.01	8.2 ± 0.3	7.13 ± 0.02	27.22 ± 0.01	30.20 ± 0.03	
**Eu(5(*S*))_3_**	1.94 ± 0.01	8.4 ± 0.3	7.18 ± 0.04	27.02 ± 0.03	30.90 ± 0.01	
**Eu(1(*R*))_3_**	0.58 ± 0.01 (18.8%)	0.6 ± 0.1	—	—	—	CH_3_OH 5.79 × 10^–5^ M
	1.95 ± 0.01 (81.2%)					
**Eu(1(*S*))_3_**	0.58 ± 0.01 (18.8%)	0.5 ± 0.1	—	—	—	
	1.95 ± 0.01 (81.2%)					
**Eu(5(*R*))_3_**	0.58 ± 0.01 (79.0%)	2.2 ± 0.2	—	—	—	
	1.76 ± 0.03 (21.0%)					
**Eu(5(*S*))_3_**	0.57 ± 0.01 (81.0%)	2.3 ± 0.1	—	—	—	
	1.58 ± 0.01 (19.0%)					
	0.57 ± 0.01 (81.0%)					

The quantum yield of the Eu(iii) complexes in CH_3_OH solution was found to be significantly lower than that seen in CH_3_CN, which we contribute to a dissociation of these complexes in more competitive protic media, where one of the ligands is removed from the **EuL_3_** complex to give **EuL_2_** + **L**. The latter now being affected by quenching of the lanthanide excited state by energy matching solvent O–H oscillators. The Eu(iii)-centred emission decays for both groups of complexes were found to be bi-exponential with the main species being **EuL_3_** (∼80%) for the complexes with **1(*S*)** and **1(*R*)** while in case of 1-naphthyl derivatives **EuL_2_** species (∼80%) were prevalent (Table 2). Interestingly, the values of *Φ*_tot_ and *τ*_obs_ between symmetrical “Trinity Sliotar”^8^ and the asymmetrical Eu(iii) complexes (**Eu(1(*S*))_3_** and **Eu(1(*R*))_3_** developed herein and that of **Eu(5(*S*))_3_** and **Eu(5(*R*))_3_**)^11^ in CH_3_CN are found to be very similar. However, if one compares the ratio between ^5^D_0_ → ^7^F_*J*_ transitions in the Eu(iii)-centred emission spectra of these two sets of complexes it is possible to identify common differences which suggest discrepancy in the Eu(iii) ion site symmetry within these two systems. In turn, *τ*_R_ of the symmetrical complexes was found to be higher as the shielding of the metal centre is better in this case compared to that seen for the asymmetrical structures. Hence, the values of *Φ*LnLn were found to be higher for asymmetrical molecules **Eu(1(*S*))_3_**, **Eu(1(*R*))_3_** and **Eu(5(*S*))_3_**, **Eu(5(*R*))_3_**, suggesting the larger influence of non-radiative processes in the symmetrical complexes. Finally, *η*_sens_ was higher for the symmetrical complexes as the asymmetrical lose one antenna per ligand. Having probed the photophysical properties of **Eu(1(*S*))_3_** and **Eu(1(*R*))_3_** we next turned our attention to analysing the role of the Eu(iii) ion in the metal directed synthesis of these complexes under kinetic control at room temperature.

### Monitoring self-assembly processes between **1(*S*)** and **1(*R*)** with Eu(iii) in CH_3_CN by absorption and luminescence spectroscopy

The self-assembly studies between **1(*S*)** or **1(*R*)** and Eu(iii) were first performed in CH_3_CN solution. However, the analysis of the data using non-linear regression analysis program SPECFIT® did not result in data convergence. As the self-assembly processes are highly dependent on the solvent and ions in solution, it was decided to introduce ionic strength into the system, as it has been shown by Piguet *et al.* ^20^ that the introduction of tetrabutylammonia perchlorate favours the formation of highly charged complexes in lanthanide-directed self-assemblies formations in aprotic solvent systems. Hence, to overcome the data convergence problem we choose to use 0.05 M tetraethylammonium chloride ((C_2_H_5_)_4_NCl) as ionic strength in our studies.

**Fig. 4:**
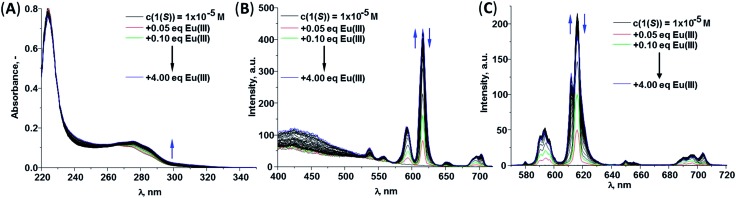
The changes in the (A) absorption, (B) fluorescence and (C) Eu(iii)-centred emission spectra of **1(*S*)** (*c* = 1 × 10^–5^ M) upon addition of Eu(CF_3_SO_3_)_3_ in CH_3_CN (25 °C, 0.05 M (C_2_H_5_)_4_NCl).

**Fig. 5:**
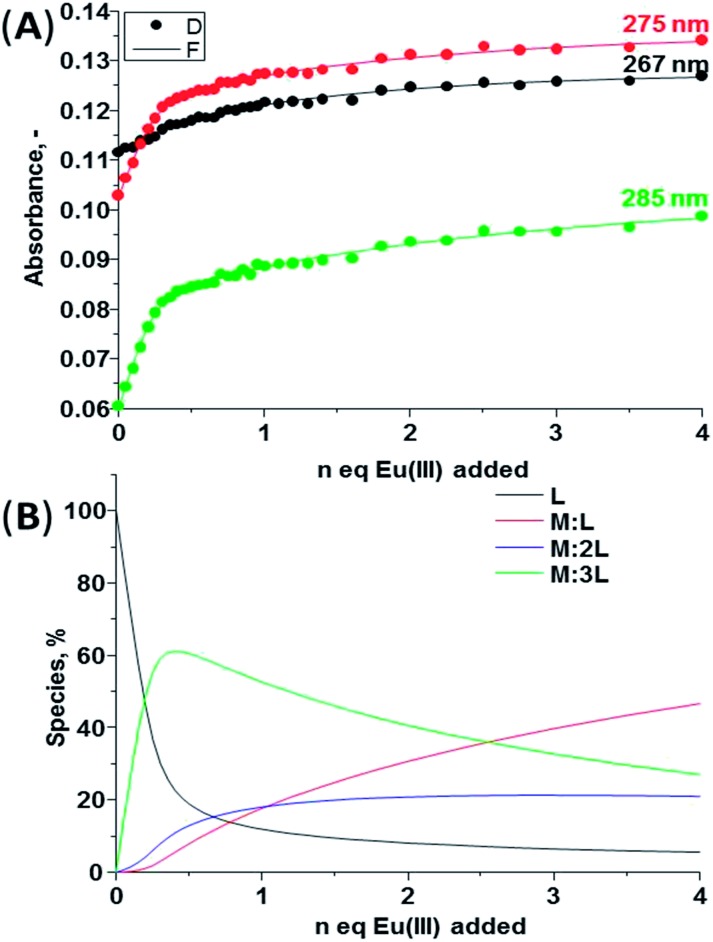
(A) Experimental binding isotherms and their corresponding fit obtained using non-linear regression analysis program SPECFIT®, (B) speciation-distribution diagram obtained from the fit of the changes in the absorption spectrum of **1(*S*)** upon addition of Eu(CF_3_SO_3_)_3_ in CH_3_CN (25 °C, 0.05 M (C_2_H_5_)_4_NCl).

The changes observed in the absorption, fluorescence and Eu(iii)-centred emission spectra upon titrating **1(*S*)** and **1(*R*)** with Eu(iii) were identical for both enantiomers and as such the discussion herein will focus only on one of these ligands. The changes seen upon titrating **1(*S*)** are shown in Fig. 4 (see ESI† for **1(*R*)**). As described previously, the absorption spectrum of **1(*S*)** possesses two main maxima at 222 and 270 nm (Fig. 4A). The addition of Eu(iii) resulted in hyperchromicity in these absorption bands, where the main changes were observed in the bands centred at ∼270 nm, as demonstrated in Fig. 5A, where the changes at three different transitions are plotted against added Eu(iii) equivalents. Here, the absorbance increase was initially observed until addition of 0.30 equivalents of Eu(iii), signifying the formation of the desired 1 : 3 stoichiometry, after which much slower increase occurred until the addition of 1.00 equivalents before beginning to plateau. The experimental changes were analysed using non-linear regression analysis program SPECFIT® (ref. 21) where factor analysis confirms the presence of four absorbing species. These were assigned to the ligand (**L**) and the Eu(iii) species **M:L**, **M:L_2_** and **M:L_3_**. The data was satisfactorily fitted to the following equilibria and the associated binding constants, expressed as log *β*_*x* : *y*_, are summarised in Table 3:**Eu** + **L** ↔ **EuL** (log *β*_1 : 1_)**Eu** + **2L** ↔ **EuL_2_** (log *β*_1 : 2_)**Eu** + **3L** ↔ **EuL_3_** (log *β*_1 : 3_)

**Table 3:** Binding constants obtained by fitting the changes in absorption, fluorescence and Eu(iii)-centred emission spectra of **1(*S*)** or **1(*R*)** upon addition of Eu(CF_3_SO_3_)_3_ in CH_3_CN solution (25 °C, 0.05 M (C_2_H_5_)_4_NCl)

Compound	Absorption			Fluorescence			Eu(iii)-centred emission		
	log *β*_1 : 1_	log *β*_1 : 2_	log *β*_1 : 3_	log *β*_1 : 1_	log *β*_1 : 2_	log *β*_1 : 3_	log *β*_1 : 1_	log *β*_1 : 2_	log *β*_1 : 3_
**1(*S*)**	5.4 ± 0.3	11.1 ± 0.6	17.3 ± 0.4	6.2 ± 0.1	12.5 ± 0.2	17.9 ± 0.2	6.7 ± 0.2	—	17.4 ± 0.4
**1(*R*)**	5.7 ± 0.2	—	16.5 ± 0.5	6.2 (fix)	—	18.1 ± 0.2	6.9 ± 0.3	—	16.4 ± 0.4

These binding constants are comparable to that seen for structurally similar self-assemblies,^11,22^ though not as high as that seen for analogues “Trinity Sliotar” complexes.^8–12^ The speciation distribution diagram for the titration is shown in Fig. 5B, which demonstrates the initial formation of 1 : 3 (**M:L**) species in *ca.* 60% yield upon addition of 0.30 equivalents of Eu(iii). However, almost simultaneously the formation of both the 1 : 2 and 1 : 1 stoichiometries also occurs. All of these species present in the solution until the end of the titration with the presence of the 1 : 1 complex in 58% yield, while the 1 : 2 and the 1 : 3 stoichiometry exist in 20% and 22% yield, respectively.

The changes in the fluorescence emission and Eu(iii)-centred emission were also monitored in parallel and are shown in Fig. 4B and C, respectively. The ligand fluorescence was weak and as such it was not possible to monitor the changes in the ligand-centred emission accurately over the cause of the titration. However, upon addition of the lanthanide to a solution of **1(*S*)** the formation of **Eu:L** assemblies between the two was clear from the appearance of the red Eu(iii)-centred emission bands due to the deactivation of ^5^D_0_ → ^7^F_*J*_ (*J* = 0–4) upon excitation of the ligand at 270 nm, Fig. 4B. Analysis of these fluorescence emission changes, showed that the luminescence intensity of the ^5^D_0_ → ^7^F_1,3,4_ bands increased up until the addition of 0.30 equivalents of Eu(iii), after which the emission plateau. In contrast, the changes in the ^5^D_0_ → ^7^F_2_ based transition were more stepwise where an initial increase was observed upon addition of 0.30 equivalents of Eu(iii), followed by slower increase in the intensity until the addition of 0.50 equivalents where the saturation of the luminescence intensity occurred.

Similar luminescence behaviour was observed in the Eu(iii)-centred emission spectra by recording the delayed emission from the ion in phosphorescence mode. Here, the fine structure in the emission transitions was more pronounced as is evident from Fig. 4C, for the splitting of ^5^D_0_ → ^7^F_2_ band into two maxima at 612 and 616 nm. The intensity of the band at 616 nm reaches its maximum upon addition of 0.30 equivalents of Eu(iii) (Fig. 4C, see ESI†) while the intensity of the band at 612 nm increases gradually until the addition of 1.00 equivalent of Eu(iii) after which the emission saturated (see ESI†). The presence of ^5^D_0_ → ^7^F_0_ band suggests the formation of 1 : 3 species with the arrangement of the ligands around Eu(iii) ion in *C*_3_ symmetry confirming the results found in the crystal structure of **Eu(1(*R*))_3_** complex (see above). Analysis of the changes in the Eu(iii)-centred emission spectra confirmed the formation of the expected 1 : 1, 1 : 2 and 1 : 3 species in the solution.

The changes in both the fluorescence and the Eu(iii)-centred emission spectra were analysed using non-linear regression analysis program SPECFIT®. As expected the factor analysis for the changes in the fluorescence emission spectra suggests the presence of four emissive species, while in the case of Eu(iii)-centred emission three emissive species were identified. The changes were fitted to the same equilibrium used for analysis of the ground state data, showing similar binding constants as seen in Table 3.

### Monitoring self-assembly formation between **1(*S*)** or **1(*R*)** and La(iii) in CD_3_CN solution by ^1^H NMR spectroscopy

**Fig. 6:**
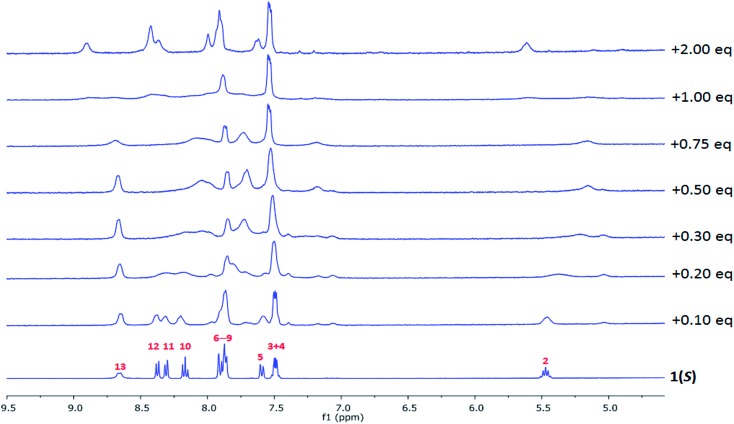
The changes in the ^1^H NMR spectra (400 MHz) of **1(*S*)** (*c* = 4.26 × 10^–4^ M) upon gradual addition of **La(CF_3_SO_3_)_3_** in CD_3_CN.

The interaction between the ligands and lanthanide ions in CD_3_CN solution was also studied using NMR spectroscopy where the binding was monitored using diamagnetic lanthanum ions as ^1^H NMR spectra of the Eu(iii) complexes were too broadened and shifted to be fully analysed. The overall changes are seen in Fig. 6, where the ^1^H NMR of the free ligand can be seen and assigned (*i.e.* Scheme 1 for assignment and ESI†).

The changes in ^1^H NMR spectra of **1(*S*)** or **1(*R*)** were followed upon addition of La(CF_3_SO_3_)_3_ and identical for both enantiomers (Fig. 6 and ESI†). This reflected the formation of a single species in solution on the NMR time-scale. Generally, upon addition of La(iii) the NMR spectra became both broadened and shifted indicating complexes formed in the solution. More specifically, the CH(**2**) protons were shown to be shifted upfield, while N–H protons (NH(**13**)) were shifted downfield. Clear binding of the La(iii) to the pyrindine ring can be also confirmed by broadening of the proton resonances (CH(**10–12**)). Similarly the changes in the naphthyl group protons CH(**3**,**4**) occurred with very minor downfield shift while CH(**5–9)** experienced much more significant changes resulting in an upfield shift and appearance of the new resonances, which is indicative of the recognition process being in slow exchange. Thus, even though the exact mode of binding cannot be established it is possible to conclude that the binding of La(iii) to **1(*S*)** or **1(*R*)** occurs through the pyridine centre with further rearrangement of the naphthyl groups around metal centre. The evolution of the changes in the spectra suggest the possible occurrence of 1 : 1, 1 : 2 and 1 : 3 (**M:L**) species.

### Circular dichroism and circularly polarised luminescence spectroscopy studies

**Fig. 7:**
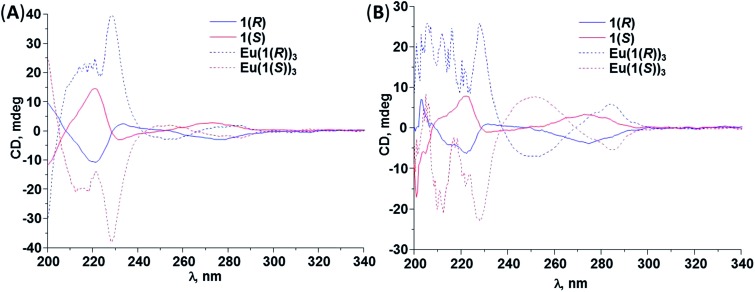
CD spectra of **1(*S*)** (–) and **1(*R*)** (–) along with **Eu(1(*S*))_3_** (---) and **Eu(1(*R*))_3_** (---) recorded in (A) CH_3_CN and (B) CH_3_OH solvents at *c* = 1 × 10^–5^ M, 25 °C.

In our previous work,^8–12^ we have used circular dichorsim and circularly polarised luminescence to demonstrate the chirality associated with the formation of lanthanide self-assemblies such as in “Trinity Sliotar” complexes, as well as triple stranded dimetallic lanthanide helicates. In these, the Eu(iii) CPL emission was recorded, demonstrating that the complexes and helicates were formed as pairs of enantiomers, where the chirality of the ligands was transferred to the complexes, giving *Δ* and *Λ* absolute stereochemistry. Similarly, we set out to probe the chiral nature and enantiomeric purity of **1(*S*)** or **1(*R*)** and their Eu(iii) complexes using CD along with CPL spectroscopy. The CD spectra of both ligands and their corresponding Eu(iii) complexes were recorded in both CH_3_OH and CH_3_CN solvent systems at 1 × 10^–5^ M and are shown in Fig. 7. All the structures showed clear Cotton effects and the expected mirror images for each pair of ligands (*i.e.* demonstrating that **1(*S*)** or **1(*R*)** are synthesised as enantiomers) and complexes in both solvent systems. The fact that both **Eu(1(*S*))_3_** and **Eu(1(*R*))_3_** give rise to equal but opposite dichroism bands is consistent with the presence of a single chiral stereoisomer in solution for these two complexes. In both CH_3_OH and CH_3_CN the CD spectra of the ligands are very similar with the π → π* transitions resulting in the signals of maxima 273, 231 and 221 nm with the only difference being the ellipticity of the signals Fig. 7, which is significantly different for the two solvent systems. This clearly demonstrates the role solvent plays herein. Moreover, the spectra of the ligands can be characterised by Davydov splitting of 11 and 42 nm and this was observed in both solvents.^14,23^ The CD spectra of **Eu(1(*S*))_3_** and **Eu(1(*R*))_3_** possess very different structure compared to the ones observed for their corresponding ligands. However, again the spectra of the complexes are very similar between CH_3_OH and CH_3_CN with the maxima at 283, 253 and 228 nm and Davydov splitting of 23 and 35 nm. It has to be noted that the ratio between the degrees of ellipticity for the maxima in the CD spectra of **EuL_3_** (**L** = **1(*S*)**, **1(*R*)**) recorded in CH_3_OH and CH_3_CN are different which can be related to the solvent effect causing dissociation of **EuL_3_** ↔ **EuL_2_** + **L** in presence of protic solvent. This was observed previously while recording the life-times of Eu(iii)-centred emission in CH_3_OH, as summarised in Table 1. The presence of only **EuL_3_** species in aprotic solvent at *c* = 1 × 10^–5^ M was confirmed by recording the Eu(iii) ^5^D_0_ excited state life-times which were best fitted to monoexponential decay with *τ* ≈ 1.99 ± 0.06 ms for both enantiomers.

The occurrence of bisignate intense CD Cotton effects suggests possible coupling between naphthyl chromophores as previously was observed by Parker *et al.* (exciton coupling).^24^ In order to elucidate the effect of temperature on the interaction of the aromatic groups we recorded CD spectra of Eu(iii) complexes in the temperature range varying from –10 to 60 °C. However, we did not observe an enhancement in the CD signals or a change in Davydov splitting (see ESI†), but the shape of CD spectra suggests the presence of the coupling interactions between aromatic antennas.

**Fig. 8:**
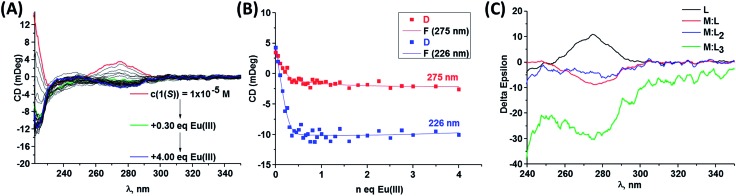
(A) Changes in CD spectra of **1(*S*)** (–) (*c* = 1 × 10^–5^ M) upon addition of Eu(CF_3_SO_3_)_3_, (B) experimental binding isotherms representing the changes in the CD bands upon gradual Eu(CF_3_SO_3_)_3_ addition in CH_3_CN (25 °C, 0.05 M (C_2_H_5_)_4_NCl) and (C) recalculated CD spectra obtained with SPECFIT®.

As stated above, CD spectroscopy has been widely used for performing quantitative analysis of various supramolecular systems, but mainly focusing on the interaction of biological substrates with organic molecules, hydrogen-bonded and salt-bridged complexes or chirality-sensing systems.^14,25^ However, to the best of our knowledge, only very few reports study the self-assembly between organic ligands and Ln(iii) ions in solution.^12b,26^ Consequently, we studied the binding equilibrium processes of Eu(iii) to both **1(*S*)** and **1(*R*)** ligands in CH_3_CN solution by monitoring the changes in the main CD bands, following a titration of these ligands with Eu(iii) as shown in Fig. 8A (see also ESI†). As the binding constants for **Eu:L_n_** assemblies were previously determined in CH_3_CN solution in presence of 0.05 M (C_2_H_5_)_4_NCl, we monitored the changes in the same ionic media. It should be stated that the observed changes for one enantiomer are mirror images of the other and this can be clearly seen from the binding isotherms of the main bands *versus* equivalents of Eu(iii) added into the solution, as shown in ESI.† In order to monitor conformational changes that can possibly occur in the solution the CD spectra were recorded directly after each addition and after 24 hours equilibration. However, in this particular case, no significant differences occurred upon equilibration. Similarly to our previous self-assembly studies the main changes in the spectra occur upon addition of 0 → 1 equivalents of Eu(iii) to the solution and indicates the formation of 1 : 1, 1 : 2 and 1 : 3 species. In order to perform more detailed analysis about the equilibria occurring in the solution here we attempted to fit the data obtained using non-linear regression analysis program SPECFIT® in a similar manner to that carried out for the changes in the ground state absorption and the emission above. For both of the enantiomers, the least square factor analysis of the titration results suggested the presence of four responding species in the CD spectra, which was in line with our previous findings discussed above. Based on our previous results and current data we anticipated the successive formation of 1 : 1, 1 : 2 and 1 : 3 species. Indeed, our analysis showed the formation of all of these species, which were comparable to those obtained by fitting the changes in the absorption and luminescence data. Hence, for example, in the case of **1(*S*)** binding constants of log *β*_1 : 1_ = 6.6 ± 0.5, log *β*_1 : 2_ = 12.8 ± 0.6 and log *β*_1 : 3_ = 18.3 ± 0.6 were determined. Gratifyingly, the fitting of the titration of **1(*R*)** with Eu(iii) gave almost indicial results (within experimental error) of log *β*_1 : 1_ = 6.5 ± 0.5, log *β*_1 : 2_ = 12.0 ± 0.8 and log *β*_1 : 3_ = 18.7 ± 0.6. These binding constant results are slightly higher than determined above, but this can be attributed to the presence of (C_2_H_5_)_4_NCl in the solution which did not allow monitoring the CD changes in the 200–223 nm spectral range and as such we were only able to analyse the changes in 275 and 226 nm bands, both of which possess small amplitude. It is clear from these results that the self-assembly processes can be both monitored and quantified, as well as that the results are comparable to that observed using more classical fitting of absorbance and emission data. Thus probing the chiroptical properties of the lanthanide directed self-assembly process in real time allows for additional information to be revealed that can help us in furthering quantification and revealing more understanding of such processes. As the overall changes in the circular dichroism spectra are quite significant they can also be employed as a fingerprint or signature for each of the stochiometries in solution. This is commonly done in the treatment of absorption spectra titrations data, where the information from the data fitting can also be employed to generate calculated spectra of each of the species in solution. The changes observed in the CD spectra of **1(*S*)** shown in Fig. 8A cannot be accurately presented as a ‘signature’ for each of the three stochiometries. In comparison, the calculated spectra generated for the changes in the CD titrations in CH_3_CN are shown in Fig. 8C for **1(*S*)** and these clearly demonstrate that each of the species can be assigned. For example, the experimental CD spectrum of **Eu(1(*S*))_3_** (Fig. 7A) shows negative band centred at 273 nm similarly to the one observed for the calculated spectrum (Fig. 8C). Each of these calculated spectra can, therefore, be employed as a fingerprint, or a signature, for that given species. This again, demonstrates the potential use of CD spectroscopy in accessing vital information about equilibrium processes in metal directed synthesis of supramolecular structures.

**Fig. 9:**
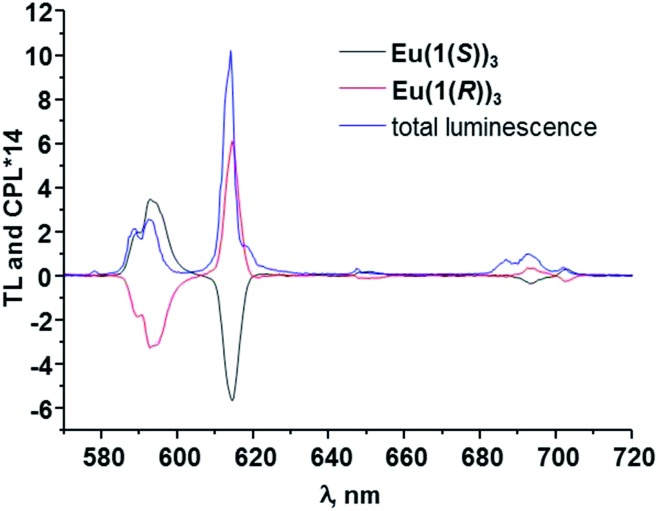
The CPL spectra for **EuL_3_** in CH_3_OH (**L** = **1(*S*)**, **1(*R*)**). The total luminescence is also shown.

Having emissive metal centre in a chiral environment we further investigated the chiroptical properties of our systems using CPL spectroscopy. As anticipated the excitation into the ligand absorption bands resulted in energy transfer to Eu(iii) ion and thus generation of mirror-image CPL spectra showing the appearance of ^5^D_0_ → ^7^F_*J*_ (*J* = 0–4) transition bands, as shown in Fig. 9. The luminescence dissymmetry factors *g*_lum_, were calculated for all of these transitions (see ESI†) and for ^5^D_0_ → ^7^F_1_ (589 nm) were found to be 0.16 and –0.15, while for ^5^D_0_ → ^7^F_2_ (614 nm) these values were equal to –0.09 for the Eu(iii) complex with **1(*S*)** and 0.10 for **1(*R*)**, respectively. These correspond well to values that we previously obtained for similar asymmetrical complexes with 1-naphthyl antennae complexes^11^ reflecting the similarities in the helical twists, nature of the ligand field, donor group solvation and time-averaged local helicity around Eu(iii) of **Eu(1(*S*))_3_** and **Eu(1(*R*))_3_**. The values for Eu(iii) complexes with asymmetrical ligands are lower than these obtained in our group previously^7,8,9b^ as well as these obtained by Muller *et al.* ^4*c*^ for the complexes with symmetrical ligands. However, this can be simply explained by the decrease in the degree of conformational rigidity of the complex when reducing the symmetry of the ligand. Overall, obtained *g*_lum_ values are high as enantiopure Eu(iii) and Tb(iii) complexes typically possess these values between 0.1 and 0.5 and much higher compared to the chiral fluorescent organic molecules where *g*_lum_ < 0.01.^3a,b^

Based on our previous work,^7,8,9b^ where we have assigned the absolute configuration of lanthanide based self-assembly complexes and helices, formed from chiral ligands with known absolute stereochemistry, as *Δ* or *Λ*, then from the circularly polarised emission in Fig. 9, we were able to do the same for **Eu(1(*S*))_3_** and **Eu(1(*R*))_3_** by comparison. The CPL spectra of both **Eu(1(*S*))_3_** and **Eu(1(*R*))_3_** were structurally identical to that observed for “Trinity Sliotar” complexes. Hence, the CPL of **Eu(1(*R*))_3_** consists of a negative CPL signal for the Δ*J* = 1, a positive band for the Δ*J* = 2, and a split CPL signal for the Δ*J* = 4 (into a positive and a negative band), and these are identical not only in their sign, but also in the intensity ratio to that seen for the *RR* isomer used in the synthesis of the *Λ* “Trinity Sliotar” complex.^7,8^ Similarly, the *RR* isomer used in the formation of structurally similar dimetallic Eu(iii) triple stranded helicates,^9*b*^ gave also such identical CPL spectra. Consequently, we can with a degree of confidence, assign the absolute stereochemistry of **Eu(1(*R*))_3_** as *Λ*; and consequently the absolute stereochemistry of the **Eu(1(*S*))_3_** complex as *Δ*. Moreover, we were able to assign the configuration of **Eu(1(*R*))_3_** with absolute certainty since we have grown its crystals of good enough quality for crystallographic determination (see above) and as such clearly relate spectroscopical data to known solid state structures of several “Trinity Sliotar” complexes resolved in our laboratory.

## Conclusions

Chiral asymmetrical *R*- and *S*- 6-(1-(naphthalen-2-yl)ethylcarbamoyl)pyridine-2-carboxylic acids (**1(*S*)** and **1(*R*)**) were obtained in three steps and high yield. These ligands were reacted with Eu(iii) ions resulting in the formation of red emissive complexes, of **Eu(1(*S*))_3_** and **Eu(1(*R*))_3_**, respectively, with ∼2% luminescence quantum yields in CH_3_CN solution. Crystal structures of **1(*S*)** and **1(*R*)** along with their benzyl protected forms (**4(*S*)** and **4(*R*)**) were obtained from CH_3_CN–CH_3_OH or CH_3_OH solvent systems and all crystallized in the chiral space group *P*2_1_, while **Eu(1(*R*))_3_** crystallized in an orthorhombic crystal system with chiral space group *C*222_1_. The self-assembly formation between **1(*S*)** and **1(*R*)** with Eu(CF_3_SO_3_)_3_ in aprotic CH_3_CN polar media were analysed using ^1^H NMR, absorption, luminescence and CD spectroscopies at room temperature. In all the cases the changes suggests successive formation of **M:L**, **M:L_2_** and **M:L_3_** assemblies with comparable values of the binding constants. As expected the excitation into the ligands absorption bands resulted in the transfer of the chirality from the ligand onto the metal centres showing characteristic Eu(iii) CPL bands. This allowed us to tentatively assign the absolute stereochemistry of the self-assemblies as *Δ* and *Λ* for **Eu(1(*S*))_3_** and **Eu(1(*R*))_3_**, respectively, and absolutely confirm it for **Eu(1(*R*))_3_** by comparing to the solid state crystallographic data obtained. Here, we represent one of the rare examples where the binding constants of supramolecular self-assemblies were determined by fitting the changes in the chiroptical spectra (CD) using non-linear regression analysis. This allowed us to identify three species in solution as the 1 : 1, 1 : 2 and 1 : 3 metal to ligand stoichiometries and quantify their binding constants, all of which gave good correlation with those determined by fitting the changes in the absorption and luminescence spectra. Moreover, the analysis of the CD spectra of the ligands and their Eu(iii) complexes allowed us to suggest the presence of exciton coupling between the aromatic chromophores in these assemblies. Furthermore, using the information of the fitting of the CD data, allowed us to calculate the CD spectra of each of the three stoichiometries, which we can use as fingerprints or signatures for each one. We are actively employing CD spectroscopy in greater detail for the analysis of metal-directed synthesis of supramolecular structures.

## Experimental

### Materials and methods

All solvents and chemicals were purchased from commercial sources and used without further purification. Dichloromethane and methanol were freshly distilled under argon atmosphere prior to use. Water was purified using a Millipore Milli-Q water purification system (18.2 MΩ cm). Hydrochloric acid, sodium bicarbonate, Na_2_SO_4_, MgSO_4_, **H_2_dpa**, benzyl bromide, HOBt, triethylamine (Et_3_N), triethylsilane (Et_3_SiH), palladium on carbon (10 wt% loading), tetraethylammonium chloride ((C_2_H_5_)_4_NCl), Eu(CF_3_SO_3_)_3_·6H_2_O were purchased from Sigma-Aldrich, while *N*,*N*-dimethylformamide, (*S*)- or (*R*)-1-(1-naphthyl)-ethylamine and (*S*)- or (*R*)-1-(2-naphthyl)-ethylamine and EDCI·HCl from TCI Europe. Deuterated solvents used for NMR analysis (CDCl_3_, CD_3_OD, (CD_3_)_2_SO) were purchased from Apollo Scientific. The ^1^H NMR spectra were recorded at 400 MHz using an Agilent Technologies 400-MR NMR Spectrometer. The ^13^C NMR spectra were recorded at 100 MHz using an Agilent Technologies 400-MR NMR Spectrometer. NMR spectra were also recorded using a Bruker AV-600 instrument operating at 600.1 MHz for ^1^H NMR and 150.9 MHz for ^13^C NMR. ^1^H NMR titrations were recorded using Bruker Spectrospin DPX-400 instrument operating at 400.1 MHz. The titrations for both enantiomers were started with the ligands at *c* = 4.26 × 10^–4^ M upon gradual addition of La(CF_3_SO_3_)_3_ solution in CD_3_CN. Chemical shifts are reported in ppm with the deuterated solvent as the internal reference. All NMR spectra were carried out at 293 K. Mass-spectrometry was carried out using HPLC grade solvents. Electrospray mass spectra were determined on a Micromass LCT spectrometer and high resolution mass spectra were determined relative to a standard of leucine enkephaline. Maldi-Q-Tof mass spectra were carried out on a MALDI-Q-TOF-Premier (Waters Corporation, Micromass MS technologies, Manchester, UK) and high resolution mass spectrometry was performed using Glu-Fib with an internal reference peak of *m*/*z* 1570.6774. Melting points were determined using an Electrothermal IA9000 digital melting point apparatus. Infrared spectra were recorded on a Perkin Elmer Spectrun One FT-IE spectrometer equipped with universal ATR sampling accessory. Elemental analysis was conducted at the Microanalytical Laboratory, School of Chemistry and Chemical Biology, University College Dublin.

Complexation reactions were carried out in 2–5 mL Biotage Microwave Vials in a Biotage Initiator Eight EXP microwave reactor.

### Crystallographic experimental section

Diffraction data for all compounds were collected on a Bruker APEX 2 DUO CCD diffractometer using graphite-monochromatized Mo-Kα (*λ* = 0.71073 Å) and Incoatec IμS Cu-Kα (*λ* = 1.54178 Å) radiation. Crystals were mounted in a cryoloop/MiTeGen micromount and collected at 100(2) K using an Oxford Cryosystems Cobra low temperature device. Data were collected using omega and phi scans and were corrected for Lorentz and polarization effects.^27*a*^

The structures **1(*R*)**, **1(*S*)**, **4(*R*)** and **4(*S*)** were solved by direct methods and refined by full-matrix least-squares procedures on *F* ^2^ using SHELXL-2013 software.^27*b*^ All non-hydrogen atoms were refined anisotropically. Hydrogen atoms were added geometrically in calculated positions and refined using a riding model.

The structure for complex **Eu(1(*R*))_3_** was solved initially using SHELXS-97 which was further refined using SHELXL-97. Some of the aromatic moieties which showed high disorder were constrained to regular geometry. Low resolution and low data/parameter ratio prevented in some cases full anisotropic refinement. The thermal parameters were either restrained or refined isotropically. Hydrogen atoms were placed geometrically using suitable constraints except for water molecules in which case they were placed to form a coherent hydrogen bond network and their positions kept fixed.

Details of the data collection and refinement are given in Table 1.†

### Photophysical measurements

Unless otherwise stated, all measurements were performed at 298 K in acetonitrile solutions (spectroscopy grade, Aldrich). UV-visible absorption spectra were measured in 1 cm quartz cuvettes on a Varian Cary 50 spectrophotometer. Baseline correction was applied for all spectra. Emission (fluorescence, phosphorescence and excitation) spectra and life-times were recorded on a Varian Cary Eclipse Fluorimeter. Quartz cells with a 1 cm path length from Hellma were used for these measurements. The temperature was kept constant throughout the measurements at 298 K by using a thermostated unit block. Phosphorescence life-times of the Eu(^5^D_0_) excited state were measured in both water/deuterated water solutions in time-resolved mode at 298 K. They are averages of three independent measurements, which were made by monitoring the emission decay at 616 nm, which corresponds to the maxima of the Eu(iii) ^5^D_0_ → ^7^F_2_ transition, enforcing a 0.1 ms delay, and were analyzed using Origin 7.5®. The number of water molecules directly bonded to Eu(iii) center (*q* value) was determined according to the equation developed by Parker *et al.*:^17^

1 *q* = *A*(*τ*_O–H_^–1^ – *τ*_O–D_^–1^)

where *τ*_O–H_ is the life-time water or methanol solutions, *τ*_O–D_ is the life-time measured in deuterated water or deuterated methanol solutions.

The quantum yields (*Q*Eu,Lrel) were measured by relative method^28,29^ using Cs_3_[Eu(**dpa**)_3_]·9H_2_O complex in 0.1 M Tris buffer (pH = 7.45) (*Q*Euabs = 24.0 ± 2.5%)^19^ as a standard with known quantum yield, to which the absorbance and emission intensity of the sample are compared according to:
2

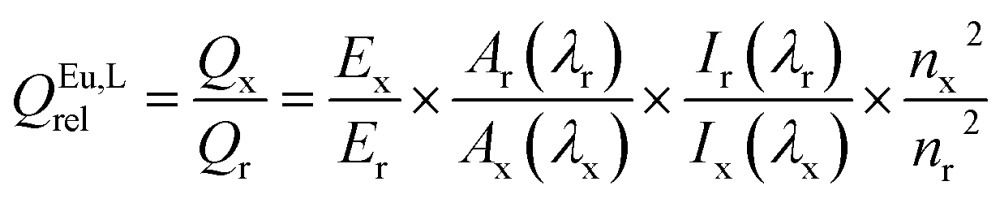

where subscript r – reference and x – sample; *E* – integrated luminescence intensity; *A* – absorbance at the excitation wavelength; *I* – intensity of the excitation light at the same wavelength, *n* – refractive index of the solution. The estimated error for quantum yields is ±10%.

*τ*_R_ life-time was obtained using eqn (3):
3

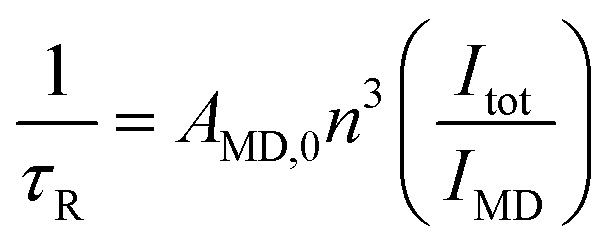

where *n* is the refractive index of the solvent, *A*_MD,0_ is the spontaneous emission probability for the ^5^D_0_ → ^7^F_1_ transition *in vacuo*, and *I*_tot_/*I*_MD_ is the ratio of the total area of the corrected Eu(iii) emission spectrum to the area of the ^5^D_0_ → ^7^F_1_ band (*A*_MD,0_ = 14.65 s^–1^).^30^

The quantum yield of the luminescence step (*Φ*LnLn) expresses how well the radiative process complete with non-radiative processes.
4

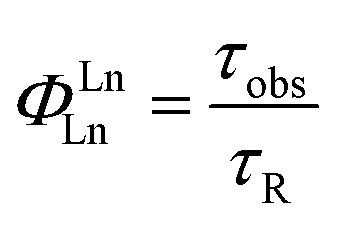




The efficiency of lanthanide sensitization (*η*_sens_) is the ratio between *Φ*_tot_ (determined experimentally) and *Φ*LnLn (see eqn (4)):
5

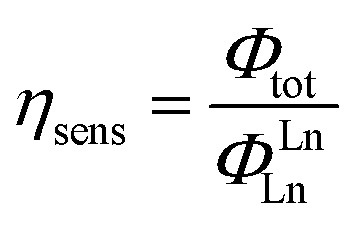




CD spectra were recorded in both acetonitrile and methanol solutions on a Jasco J-810-150S spectropolarimeter. CD titrations were performed in CH_3_CN media starting with the ligands at *c* = 1 × 10^–5^ M upon gradual addition from 0 to 4 equivalents of Eu(CF_3_SO_3_)_3_ to the solution. CPL spectra were recorded by Dr R. Peacock at the University of Glasgow. Excitation of Eu(iii) (560–581 nm) was accomplished by using a Coherent 599 tunable dye laser (0.03 nm resolution) with argon ion laser as a pump source. Calibration of the emission monochromator was accomplished by passing scattered light from a low power HeNe laser through the detection system. The optical detection system consisted of a photoelastic modulator (PEM, Hinds Int.) operating at 50 kHz and a linear polarizer, which together act as a circular analyzer, followed by a long pass filter, focusing lens and a 0.22 m double monochromator. The emitted light was detected by a cooled EM1-9558QB photomultiplier tube operating in photon counting mode. The 50 kHz reference signal from the photoelastic modulator was used to direct the incoming pulses into two separated counters. An up counter, which counts every photon pulse and thus is a measure of the total luminescence signal *I* = *I*_left_ + *I*_right_, and an up/down counter, which adds pulses when the analyzer is transmitting to the left circularly polarized light and subtracts pulses when the analyzer is transmitting right circularly polarized light. The second counter provides a measure of the differential emission intensity Δ*I* = *I*_left_ – *I*_right_.

### Spectrophotometric titrations and binding constants

The formation of the luminescent 1 : 1, 1 : 2 and 1 : 3 (**M:L**, where **M** = Eu(iii) and **L** = **1(*S*)**, **1(*R*)**) species was ascertained by both UV-visible, luminescence and CD titrations of a solution of **L** (1 × 10^–5^ M) in CH_3_CN in presence of 0.05 M (C_2_H_5_)_4_NCl with Eu(CF_3_SO_3_)_3_·6H_2_O (0 → 4 equivalents). The data were fitted using the non-linear regression analysis program, SPECFIT®.^21^

### Synthesis of 6-((benzyloxy)carbonyl)pyridine-2-carboxylic acid (**2**)

Compound **2** was synthesised by stirring 2,6-pyridinedicarboxylic acid (**H_2_dpa**; 6.00 g, 3.59 × 10^–2^ mol, 1 equivalent) with NaHCO_3_ (3.62 g, 4.31 × 10^–2^ mol, 1.2 equivalents) in *N*,*N*-dimethylformamide solution (100 mL) at 60 °C under argon for 30 minutes. Benzyl bromide (5 mL, 4.31 × 10^–2^ mol, 1.2 equivalents) was then added to this white suspension and left to stir under argon at 60 °C overnight. Resulting clear yellow solution was diluted with water and neutralised with saturated solution of NaHCO_3_ (pH ∼ 8) and extracted with diethyl ether to remove diester side product. The aqueous layer was acidified with 2 M HCl and extracted with ethyl acetate. Then, the organic layer was dried over Na_2_SO_4_, filtered and after the solvent was evaporated the resulting solid was solubilised in dichlorometane. This solution was washed with water and brine (3 × 20 mL) after which the organic layer was dried over MgSO_4_, filtered and after subsequent evaporation of the solvent white powder was obtained (3.47 g, 38% yield). M.p. 138 °C; HRMS (*m*/*z*) (ES^–^) calculated for C_14_H_10_NO_4_ *m*/*z* = 256.0610 [M – H]^–^. Found *m*/*z* = 256.0613; ^1^H NMR (400 MHz, CDCl_3_, *δ*_H_) ppm 8.39 (q, *J* = 8.40 Hz, 2H, C*H*_py_), 8.11 (t, *J* = 8.10 Hz, 1H, C*H*_py_), 7.49–7.47 (m, 2H, C*H*_ph_), 7.44–7.36 (m, 3H, C*H*_ph_), 5.46 (s, 2H, C*H*_2_); ^13^C NMR (150 MHz, CDCl_3_, *δ*_C_) 163.46, 163.39, 146.72, 139.62, 134.96, 128.85, 128.77, 128.59, 126.85, 68.07; IR *ν*_max_ (cm^–1^): 3064, 2879, 2560, 1736, 1695, 1609, 1466, 1416, 1376, 1328, 1289, 1244, 1152, 1005, 994, 956, 940, 855, 796, 784, 754, 728, 710, 648; anal. calc. for C_14_H_11_NO_4_, %: C 65.4, H 4.3, N 5.4; found, %: C 65.1, H 4.2, N 5.3.

### Preparation of **4(*S*)** and **4(*R*)**

#### General procedure

To a stirred solution of (*S* or *R*)-2-(1-aminoethyl)naphthalene (**3(*S*)** or **3(*R*)**, 1.0 equivalent) in 30 mL of freshly distilled dichloromethane, HOBt (1.0 equivalent) and 6-((benzyloxy)carbonyl)pyridine-2-carboxylic acid (**2**, 1.0 equivalent) were added. The solution was stirred for 30 minutes at 0 °C under an inert atmosphere of argon before the solution of 1-(3-dimethylaminopropyl)-3-ethylcarbodiimide hydrochloride (EDCI·HCl, 1.1 equivalents) and triethylamine (1.1 equivalents) in 30 mL of dichloromethane was added dropwise. The reaction mixture was then stirred for further 30 minutes at 0 °C under argon atmosphere, then allowed to reach room temperature and left stirring for 48 hours. The insoluble residue was removed by suction filtration before reaction mixture was washed with 1 M HCl, a saturated solution of NaHCO_3_, and water (each 2 × 20 mL). The organic layer was then dried over MgSO_4_, filtered, and the solvent removed under reduced pressure. The obtained yellow-white powders where purified using normal phase silica column chromatography eluting with 95% dichloromethane and 5% methanol (*R*_f_ = 0.9).

#### (*S*)-6-(1-(Naphthalen-2-yl)ethylcarbamoyl)pyridine-2-carboxylate (**4(*S*)**)

Compound **4(*S*)** was synthesized by stirring solution of (*S*)-1-(2-naphthyl)-ethylamine (**3(*S*)**, 0.67 g, 3.9 × 10^–3^ mol) in 30 mL of freshly distilled dichloromethane, HOBt (0.53 g, 3.9 × 10^–3^ mol) and 6-((benzyloxy)carbonyl)pyridine-2-carboxylic acid (**2**, 1.00 g, 3.9 × 10^–3^ mol) for 30 minutes at 0 °C under an inert atmosphere of argon before EDCI·HCl (0.79 g, 4.1 × 10^–3^ mol) and triethylamine (0.73 mL, 4.1 × 10^–3^ M) were then added dropwise according to the general procedure. The obtained light yellow powder was purified using normal phase silica chromatography using 95% dichloromethane–5% methanol solvent mixture as eluent (*R*_f_ = 0.9, 1.23 g, 78% yield). M.p. 130 °C; HRMS (*m*/*z*) (ES^+^) calculated for C_26_H_22_N_2_O_3_Na *m*/*z* = 433.1528 [M + Na]^+^. Found *m*/*z* = 433.1548; ^1^H NMR (400 MHz, CDCl_3_, *δ*_H_) ppm 8.50 (d, *J* = 8.50 Hz, 1H, N*H*), 8.39 (d, *J* = 8.38 Hz, 1H, C*H*), 8.23 (d, *J* = 8.23 Hz, 1H, C*H*), 7.99 (t, *J* = 8.00 Hz, 1H, C*H*), 7.85–7.81 (m, 4H, C*H*), 7.53 (d, *J* = 7.52 Hz, 1H, C*H*), 7.47 (m, 4H, C*H*), 7.34 (m, 3H, C*H*), 5.50 (t, *J* = 5.52 Hz, 1H, C*H*), 5.44 (s, 2H, C*H*_2_), 1.72 (d, *J* = 6.92 Hz, 3H, C*H*_3_); ^13^C NMR (100 MHz, CDCl_3_, *δ*_C_) 164.34, 162.76, 150.31, 146.67, 140.58, 138.64, 135.55, 133.50, 132.89, 128.78, 128.68, 128.61, 128.34, 128.10, 127.73, 127.44, 126.30, 125.98, 125.60, 124.80, 124.78, 67.64, 49.27, 22.11; IR *ν*_max_ (cm^–1^): 3396, 3060, 1732, 1680, 1588, 1504, 1452, 1439, 1381, 1283, 1229, 1182, 1165, 1151, 1083, 991, 959, 928, 897, 862, 770, 714, 689, 663, 649; anal. calc. for C_26_H_22_N_2_O_3_, %: C 76.1, H 5.4, N 6.8; found, %: C 75.9, H 5.3, N 6.7.

#### Benzyl-(*R*)-6-(1-(naphthalen-2-yl)ethylcarbamoyl)pyridine-2-carboxylate (**4(*R*)**)

Compound **4(*R*)** was synthesized by stirring solution of (*R*)-1-(2-naphthyl)-ethylamine (**3(*R*)**, 0.67 g, 3.9 × 10^–3^ mol) in 30 mL of freshly distilled dichloromethane, HOBt (0.53 g, 3.9 × 10^–3^ mol) and 6-((benzyloxy)carbonyl)pyridine-2-carboxylic acid (**2**, 1.00 g, 3.9 × 10^–3^ mol) for 30 minutes at 0 °C under an inert atmosphere of argon before EDCI·HCl (0.79 g, 4.1 × 10^–3^ mol) and triethylamine (0.73 mL, 4.1 × 10^–3^ M) were then added dropwise according to the general procedure. The obtained light yellow powder was purified using normal phase silica chromatography using 95% dichloromethane–5% methanol solvent mixture as eluent (*R*_f_ = 0.9, 1.30 g, 81% yield). M.p. 130 °C; HRMS (*m*/*z*) (ES^+^) calculated for C_26_H_22_N_2_O_3_Na *m*/*z* = 433.1528 [M + Na]^+^. Found *m*/*z* = 433.1529; ^1^H NMR (400 MHz, CDCl_3_, *δ*_H_) ppm 8.50 (d, *J* = 8.50 Hz, 1H, N*H*), 8.39 (d, *J* = 8.39 Hz, 1H, C*H*), 8.23 (d, *J* = 8.23 Hz, 1H, C*H*), 7.99 (t, *J* = 8.00 Hz, 1H, C*H*), 7.85–7.80 (m, 4H, C*H*), 7.53 (d, *J* = 7.53 Hz, 1H, C*H*), 7.47 (m, 4H, C*H*), 7.34 (m, 3H, C*H*), 5.50 (t, *J* = 5.50 Hz, 1H, C*H*), 5.44 (s, 2H, C*H*_2_), 1.72 (d, *J* = 7.04 Hz, 3H, C*H*_3_); ^13^C NMR (100 MHz, CDCl_3_, *δ*_C_) 164.37, 162.78, 150.34, 146.69, 140.60, 138.66, 135.56, 133.52, 132.91, 128.80, 128.69, 128.63, 128.35, 128.12, 127.74, 127.45, 126.31, 125.99, 125.63, 124.82, 124.79, 67.66, 49.29, 22.13; IR *ν*_max_ (cm^–1^): 3396, 1732, 1681, 1588, 1501, 1453, 1439, 1381, 1282, 1229, 1165, 1151, 1084, 991, 959, 897, 862, 842, 815, 749, 731, 689, 662, 649; anal. calc. for C_26_H_22_N_2_O_3_, %: C 76.1, H 5.4, N 6.8; found, %: C 75.8, H 5.4, N 6.5.

### Preparation of **1(*S*)** and **1(*R*)** ^16^

#### General procedure

To a stirred solution of **4(*S*)** or **4(*R*)** (1 equivalent) and 10 wt% Pd–C (10–20% by weight) in methanol was added neat triethylsilane (20 equivalents) dropwise from a pressure-equalizing dropping funnel under an argon-filled balloon. The completion of the reaction was monitored using neutral phase silica TLC plates (95% dichloromethane–5% methanol). After the reaction was complete the solvent was evaporated and resulting oily product was taken into saturated solution of NaHCO_3_. The aqueous layer was washed with diethyl ether in order to remove possible residue of starting material and triethylsilane and acidified using 2 M HCl until pH ∼ 2 when a white precipitate occurred in the solution. The solid product was washed out of the aqueous layer with ethyl acetate which was then dried over Na_2_SO_4_ and filtered. The solvent was then evaporated under reduced pressure yielding the product as white polycrystalline precipitate.

#### (*S*)-6-(1-(Naphthalen-2-yl)ethylcarbamoyl)pyridine-2-carboxylic acid (**1(*S*)**)

Triethylsilane (5.23 mL, 3.27 × 10^–2^ mol, 20 equivalents) solution in 10 mL of methanol was added dropwise to the solution of **4(*S*)** (0.67 g, 1.64 × 10^–3^ mol, 1 equivalent) and 10 wt% Pd–C (0.13 g) in 20 mL of methanol. After the workup described in the general procedure white crystalline powder was obtained (0.40 g, 77% yield). M.p. 115 °C; HRMS (*m*/*z*) (ES^–^) calculated for C_19_H_15_N_2_O_3_ *m*/*z* = 319.1083 [M – H]^–^. Found *m*/*z* = 319.1075; ^1^H NMR (600 MHz, CD_3_OD, *δ*_H_) ppm 8.32 (d, *J* = 8.32 Hz, 2H, C*H*_py_), 8.16 (t, *J* = 8.17 Hz, 1H, C*H*_py_), 7.89 (s, 1H, C*H*_naph_), 7.83 (m, 3H, C*H*_naph_), 7.58 (dd, ^3^ *J*_H–H_ = 7.58 Hz, ^4^ *J*_H–H_ = 7.59 Hz, 1H, C*H*_naph_), 7.44 (m, 2H, C*H*_naph_), 5.44 (q, *J* = 5.46 Hz, 1H, C*H*), 1.73 (d, *J* = 7.04 Hz, 3H, C*H*_3_); ^13^C NMR (150 MHz, CD_3_OD, *δ*_C_) 167.89, 164.89, 151.39, 148.35, 142.61, 140.58, 134.84, 134.06, 129.32, 128.91, 128.60, 128.57, 127.15, 126.84, 126.78, 125.86, 125.64, 50.73, 22.09; IR *ν*_max_ (cm^–1^): 3291, 3058, 2978, 2930, 1732, 1654, 1527, 1451, 1347, 1243, 1180, 999, 952, 856, 818, 745, 681; anal. calc. for C_19_H_10_N_2_O_3_·0.1CH_2_Cl_2_, %: C 69.8, H 4.9, N 8.5; found, %: C 69.9, H 4.8, N 8.4.

#### (*R*)-6-(1-(Naphthalen-2-yl)ethylcarbamoyl)pyridine-2-carboxylic acid (**1(*R*)**)

Triethylsilane (7.78 mL, 4.87 × 10^–2^ mol, 20 equivalents) solution in 10 mL of methanol was added dropwise to the solution of **4(*R*)** (1.0 g, 2.44 × 10^–3^ mol, 1 equivalent) and 10 wt% Pd–C (0.2 g) in 20 mL of methanol. After the workup described in the general procedure white crystalline powder was obtained (0.62 g, 80% yield). M.p. 115 °C; HRMS (*m*/*z*) (ES^–^) calculated for C_19_H_15_N_2_O_3_ *m*/*z* = 319.1083 [M – H]^–^. Found *m*/*z* = 319.1080; ^1^H NMR (600 MHz, CD_3_OD, *δ*_H_) ppm 8.32 (dd, ^3^J_H–H_ = 8.32 Hz, ^4^J_H–H_ = 8.33 Hz, 2H, CH_py_), 8.15 (t, *J* = 8.16 Hz, 1H, CH_py_), 7.88 (s, 1H, CH_naph_), 7.82 (m, 3H, CH_naph_), 7.58 (dd, ^3^ *J*_H–H_ = 7.58 Hz, ^4^ *J*_H–H_ = 7.59 Hz, 1H, CH_naph_), 7.44 (m, 2H, CH_naph_), 5.45 (q, *J* = 5.46 Hz, 1H, CH), 1.72 (d, *J* = 7.06 Hz, 3H, CH_3_); ^13^C NMR (150 MHz, CD_3_OD, *δ*_C_) 167.66, 164.93, 151.43, 148.06, 142.39, 140.54, 134.91, 134.21, 129.32, 128.90, 128.60, 128.58, 127.15, 126.87, 126.78, 125.85, 125.64, 50.73, 22.14; IR *ν*_max_ (cm^–1^): 3285, 3062, 2980, 2933, 1751, 1650, 1524, 1451, 1334, 1252, 1184, 952, 892, 847, 817, 743, 678, 642; anal. calc. for C_19_H_10_N_2_O_3_·0.1CH_2_Cl_2_, %: C 69.8, H 4.9, N 8.5; found, %: C 69.9, H 4.7, N 8.4.

### General synthesis of europium complexes

Eu(iii) complexes were prepared by refluxing, under microwave radiation, the relevant ligand with Eu(CF_3_SO_3_)_3_·6H_2_O (0.33 equiv.) in acetonitrile (15 mL) for 30 minutes. The solution was subsequently cooled to room temperature and then precipitated by slow evaporation of the solvent at ambient conditions. The resulting white solid was filtered off and dried under vacuum.

#### Synthesis of** Eu(1(*S*))_3_**

This complex was synthesized according to general procedure using ligand **1(*S*)** (0.060 g, 1.83 × 10^–4^ mol) and Eu(CF_3_SO_3_)_3_·6H_2_O (0.037 g, 6.10 × 10^–5^ mol). A white solid was obtained (0.035 g, 36.7% yield). Compound decomposed within 140–160 °C; HRMS (*m*/*z*) (MALDI-MS^+^) calculated for C_57_H_45_N_6_O_9_EuK *m*/*z* = 1147.2084 [Eu(**1(*S*)**–H^+^)_3_ + K^+^]^+^; found *m*/*z* = 1147.2034; ^1^H NMR (600 MHz, CD_3_CN, *δ*_H_) ppm 9.19, 8.67, 8.54, 8.30, 8.19, 8.07, 7.87, 7.57, 7.50, 7.33, 7.25, 7.09, 5.43, 5.29, 4.69, 4.56, 4.32, 3.96, 3.50, 3.05, 2.94, 2.73, 1.70, 1.27, 0.88, 0.43, 0.14, –0.10, –0.75; IR *ν*_max_ (cm^–1^): 3271, 3105, 1626, 1592, 1560, 1451, 1353, 1279, 1241, 1160, 1092, 1028, 905, 860, 820, 751, 707, 661, 635, 570, 547, 532, 523; anal. calc. for 1.0[EuC_60_H_48_F_9_N_6_O_18_S_3_]·1.3[EuC_57_H_45_N_6_O_9_] (**[Eu(1(*S*))_3_(CF_3_SO_3_)_3_]·1.3[Eu(1(*S*)–H)_3_]**), %: C 53.8, H 3.2, N 6.4, S 3.2; found, %: C 53.6, H 3.6, N 6.4, S 3.2.

#### Synthesis of **Eu(1(*R*))_3_**

This complex was synthesized according to general procedure using ligand **1(*R*)** (0.061 g, 1.86 × 10^–4^ mol) and Eu(CF_3_SO_3_)_3_·6H_2_O (0.04 g, 6.18 × 10^–5^ mol). A white solid was obtained (0.032 g, 33.3% yield). Compound decomposed within 140–160 °C; HRMS (*m*/*z*) (MALDI-MS^+^) calculated for C_57_H_45_N_6_O_9_EuK *m*/*z* = 1147.2084 [Eu(1(*R*)–H^+^)_3_ + K^+^]^+^; found *m*/*z* = 1147.2039; ^1^H NMR (600 MHz, CD_3_CN, *δ*_H_) ppm 9.19, 8.66, 8.32, 8.05, 7.86, 7.55, 7.25, 7.09, 6.30, 5.28, 4.67, 4.53, 4.30, 3.60, 3.08, 2.96, 2.72, 1.94, 1.70, 1.27, 0.88, 0.44, 0.04, –0.68, –1.27; IR *ν*_max_ (cm^–1^): 3269, 3098, 1625, 1591, 1560, 1451, 1352, 1279, 1242, 1224, 1158, 1092, 1028, 905, 860, 820, 750, 732, 680, 661, 635, 575, 554, 542, 525; anal. calc. for 1.0[EuC_60_H_48_F_9_N_6_O_18_S_3_]·1.3[EuC_57_H_45_N_6_O_9_] (**[Eu(1(*R*))_3_(CF_3_SO_3_)_3_]·1.3[Eu(1(*R*)–H)_3_]**), %: C 53.8, H 3.2, N 6.4, S 3.4; found, %: C 53.6, H 3.6, N 6.4, S 3.2.

## References

[cit1] Heffern M. C., Matosziuk L. M., Meade T. J. (2014). Chem. Rev..

[cit2] Pazos E., Vázquez M. E. (2014). Biotechnol. J..

[cit3] Muller G. (2009). Dalton Trans..

[cit4] Cantuel M., Bernardinelli G., Muller G., Riehl J. P., Piguet C. (2004). Inorg. Chem..

[cit5] Bradberry S. J., Savyasachi A. J., Martínez-Calvo M., Gunnlaugsson T. (2014). Coord. Chem. Rev..

[cit6] Gassner A.-L., Duhot C., Bünzli J.-C. G., Chauvin A.-S. (2008). Inorg. Chem..

[cit7] Leonard J. P., Jensen P., McCabe T., O'Brien J. E., Peacock R. D., Kruger P. E., Gunnlaugsson T. (2007). J. Am. Chem. Soc..

[cit8] Kotova O., Kitchen J. A., Lincheneau C., Peacock R. D., Gunnlaugsson T. (2013). Chem.–Eur. J..

[cit9] Comby S., Stomeo F., McCoy C. P., Gunnlaugsson T. (2009). Helv. Chim. Acta.

[cit10] Lincheneau C., Leonard J. P., McCabe T., Gunnlaugsson T. (2011). Chem. Commun..

[cit11] Lincheneau C., Destribats C., Barry D. E., Kitchen J. A., Peacock R. D., Gunnlaugsson T. (2011). Dalton Trans..

[cit12] Kitchen J. A., Barry D. E., Mercs L., Albrecht M., Peacock R. D., Gunnlaugsson T. (2012). Angew. Chem., Int. Ed..

[cit13] Shavaleev N. M., Gumy F., Scopelliti R., Bünzli J.-C. G. (2009). Inorg. Chem..

[cit14] Pescitelli G., Di Bari L., Berova N. (2014). Chem. Soc. Rev..

[cit15] Hamuro Y., Geib S. J., Hamilton A. D. (1997). J. Am. Chem. Soc..

[cit16] Mandal P. K., McMurray J. S. (2007). J. Org. Chem..

[cit17] Beeby A., Clarkson I. M., Dickins R. S., Faulkner S., Parker D., Royle L., de Sousa A. S., Williams J. A. G., Woods M. (1999). J. Chem. Soc., Perkin Trans. 2.

[cit18] Cho C.-W., Krische M. J. (2006). Org. Lett..

[cit19] Chauvin A.-S. Gumy F. Imbert D. Bünzli J.-C. G., Spectrosc. Lett., 2004, 375 , 517 –532 , ; *Spectrosc. Lett.*, 2007, **40**, 193 .

[cit20] Canard G., Koeller S., Bernardinelli G., Piguet C. (2008). J. Am. Chem. Soc..

[cit21] Gampp H., Maeder M., Meyer C. J., Zuberbühler A. D. (1986). Talanta.

[cit22] Le Borgne T., Altmann P., André N., Bünzli J.-C. G., Bernardinelli G., Morgantini P.-Y., Weber J., Piguet C. (2004). Dalton Trans..

[cit23] Berova N., Di Bari L., Pescitelli G. (2007). Chem. Soc. Rev..

[cit24] Dickins R. S., Howard J. A. K., Moloney J. M., Parker D., Peacock R. D., Siligardi G. (1997). Chem. Commun..

[cit25] Comprehensive chiroptical spectroscopy, ed. N. Berova, P. L. Polavarapu, K. Nakanishi and R. W. Woody, Wiley-VCH, New York, 2012.

[cit26] Telfer S. G., Tajima N., Kuroda R., Cantuel M., Piguet C. (2004). Inorg. Chem..

[cit27] (a) Bruker APEX v2012.12–0, Bruker AXS Inc., Madison, Wisconsin, USA, 2012.

[cit28] de Sá G. F., Nunez L., Wang Z. M., Choppin G. R. (1993). J. Alloys Compd..

[cit29] Demas J. N., Crosby G. A. (1971). J. Phys. Chem..

[cit30] Werts M. H. V., Jukes R. T. F., Verhoven J. W. (2002). Phys. Chem. Chem. Phys..

