# Supplementary material for: The application of chiroptical spectroscopy (circular dichroism) in quantifying binding events in lanthanide directed synthesis of chiral luminescent self-assembly structures
Source: Chem Sci. 2014 Oct 10;6(1):457–71. doi: 10.1039/c4sc02474e (PMC5588118; doi:10.1039/c4sc02474e)

Electronic Supplementary Material (ESI) for Chemical Science.
This journal is © The Royal Society of Chemistry 2014

The application of chiroptical spectroscopy (circular dichroism) in quantifying binding events in lanthanide directed synthesis of chiral luminescent self-assembly structures

Oxana Kotova,[a]* Salvador Blasco,[a] Brendan Twamley,[a] John O'Brien,[a]
Robert D. Peacock,[b] Jonathan A. Kitchen,[a,c] Miguel Martinez-Calvo[a] and Thorfinnur Gunnlaugsson[a]*

Scheme S1. Schematic representation of the ligands **1(*S*)**, **1(*R*)** and **5(*S*)**, **5(*R*)** along with their corresponding Eu(III) complexes.

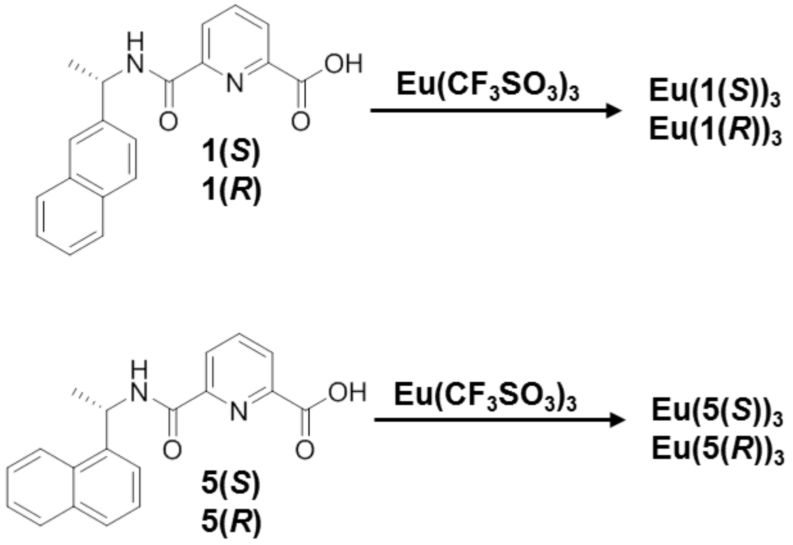

Figure S1. ^{1}H NMR of **2** (400 MHz, $CDCl_3$).

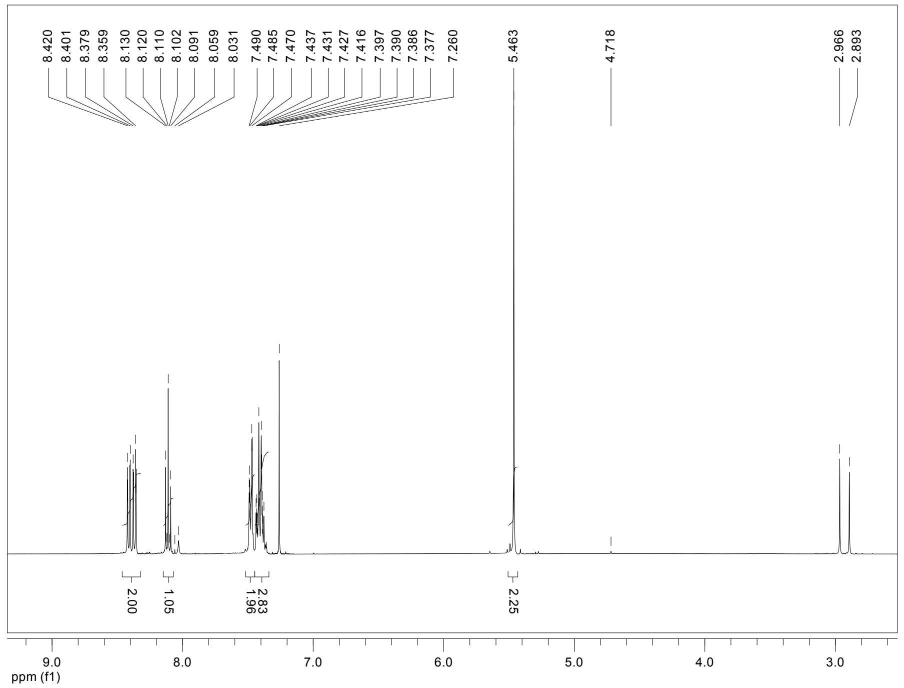

Figure S2. ^{13}C NMR of **2** (100 MHz, $CDCl_3$).

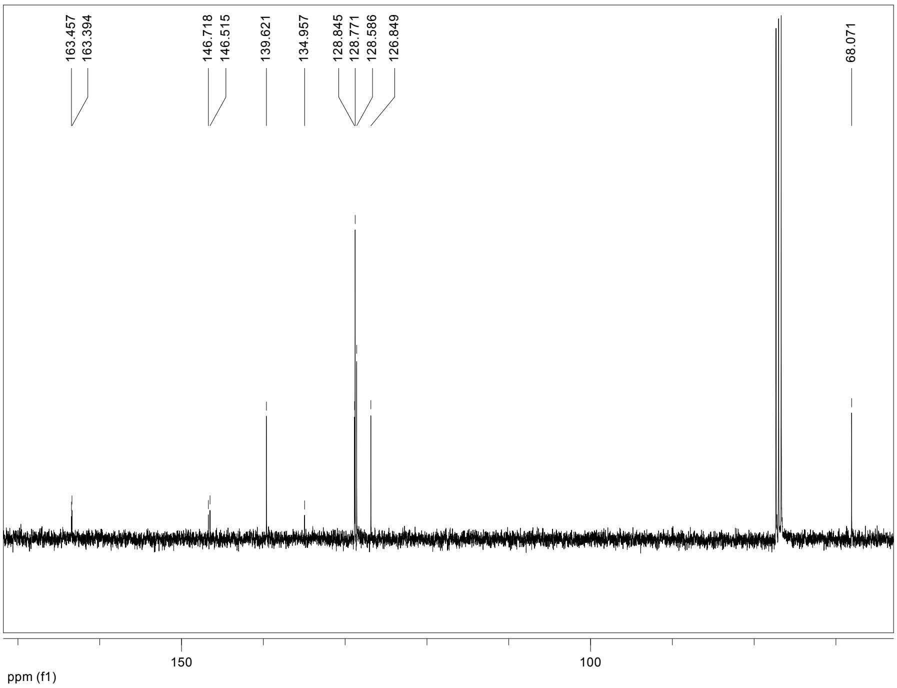

Figure S3. ^{1}H NMR of **4(*S*)** (400 MHz, $CDCl_3$).

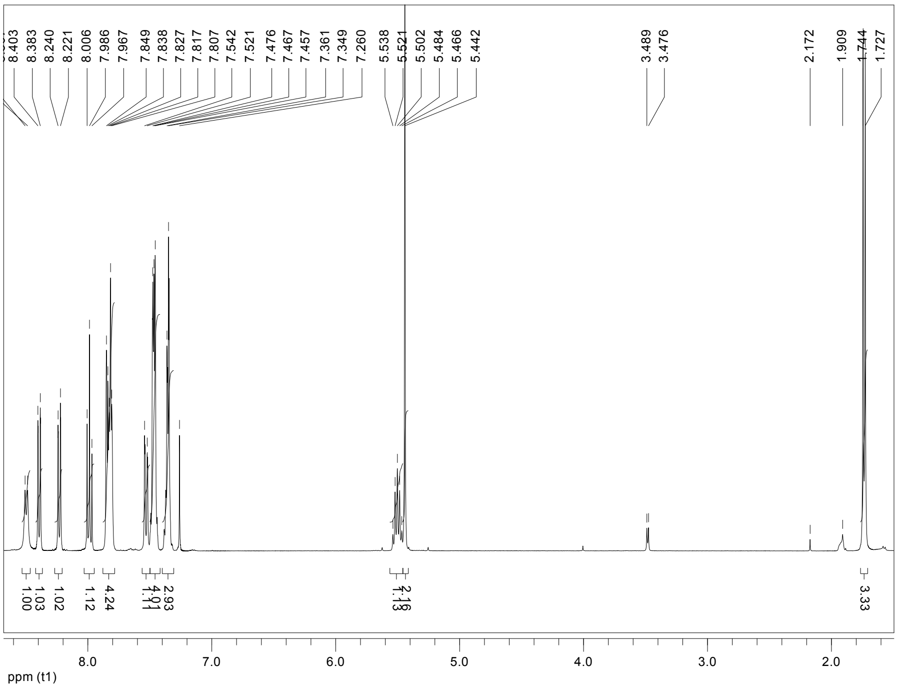

Figure S4. ^{13}C NMR of **4(*S*)** (100 MHz, $CDCl_3$).

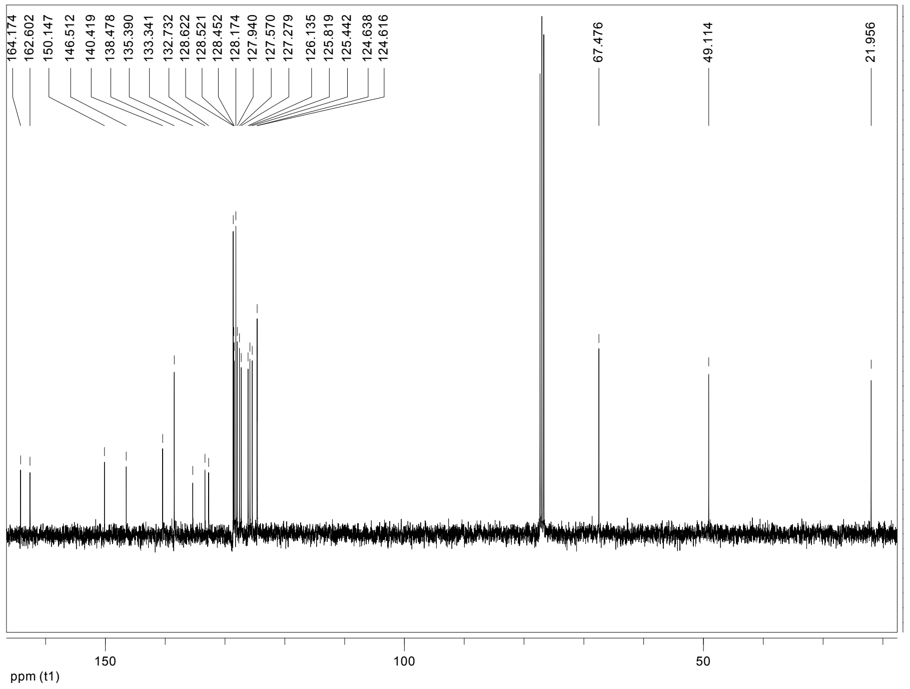

Figure S5. ^{1}H NMR of **4(*R*)** (400 MHz, $CDCl_3$).

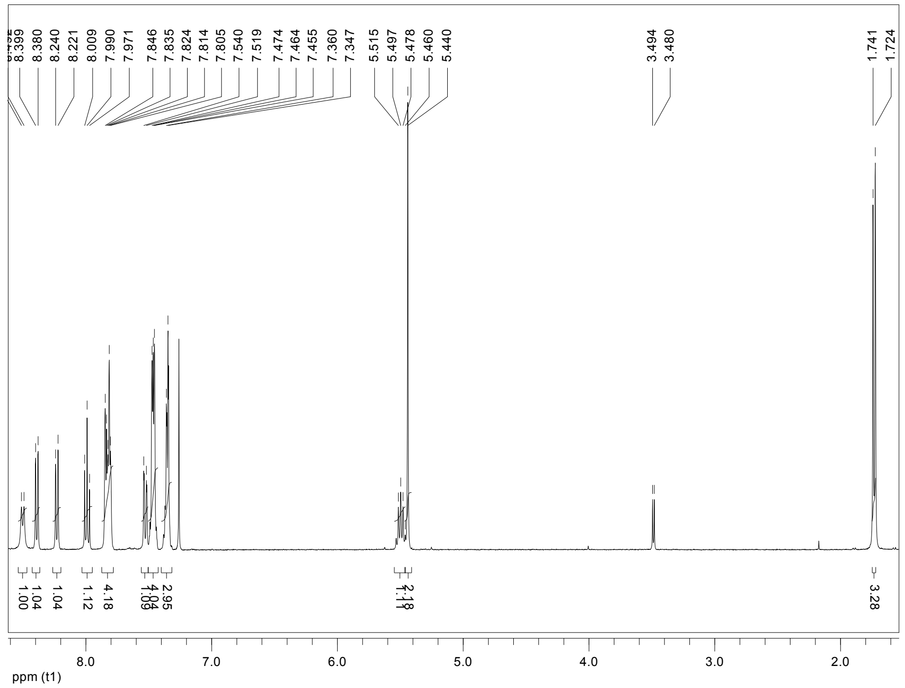

Figure S6. ^{13}C NMR of **4(*R*)** (100 MHz, $CDCl_3$).

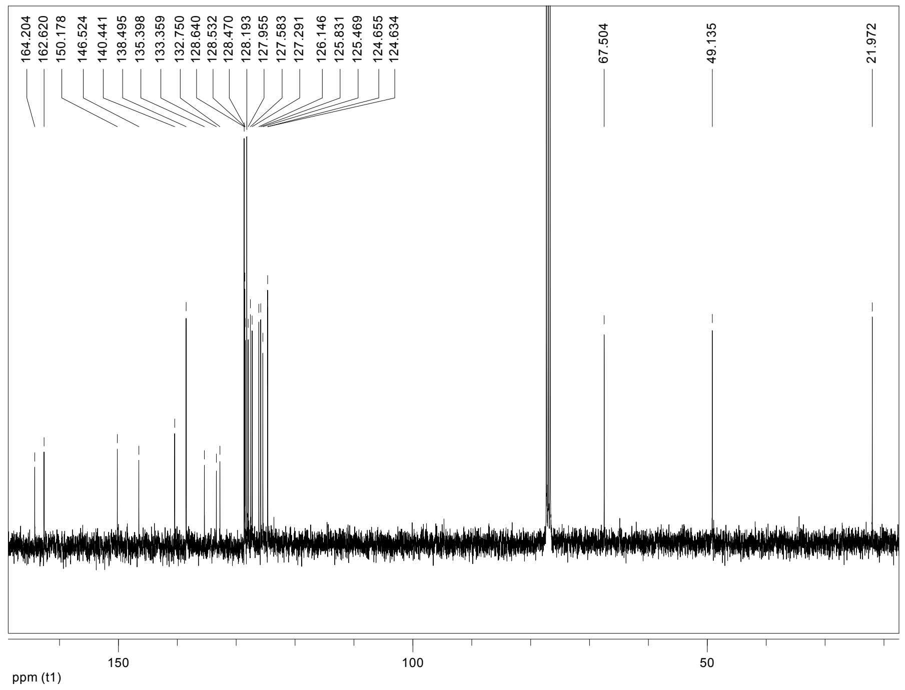

Figure S7. ^{1}H NMR of **1(*R*)** (600 MHz, CD_3OD).

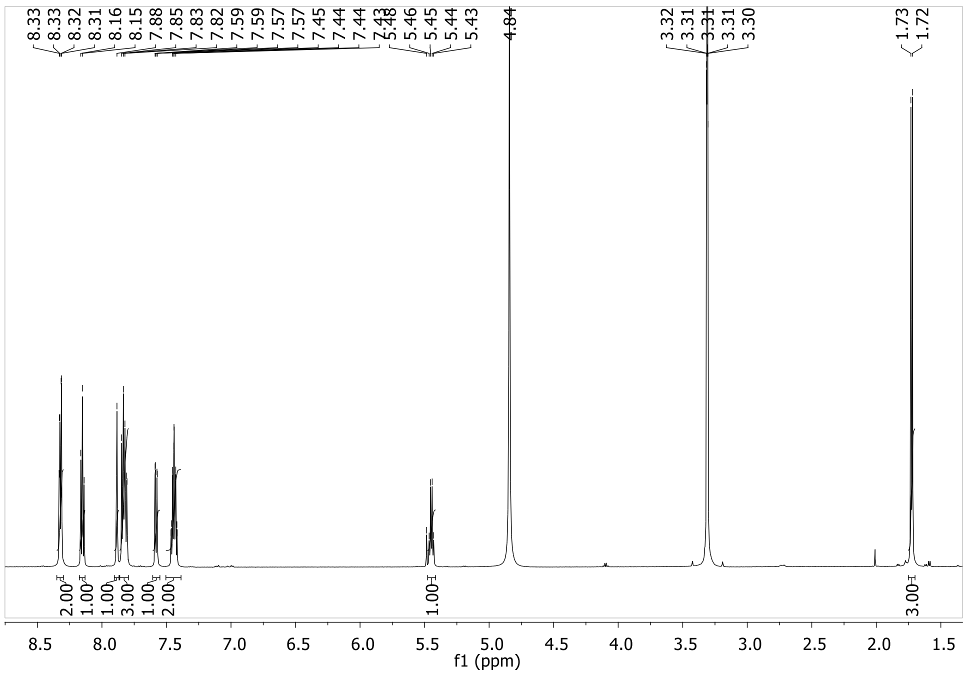

Figure S8. ^{13}C NMR of **1(*R*)** (150 MHz, CD_3OD).

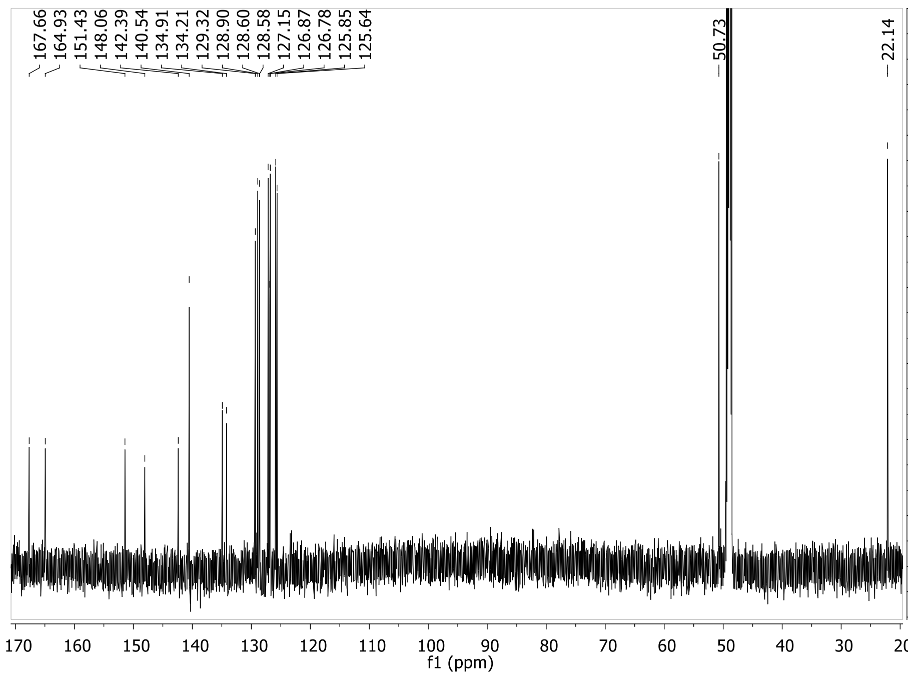

Figure S9. ^{13}C NMR of **1(*S*)** (150 MHz, CD_3OD).

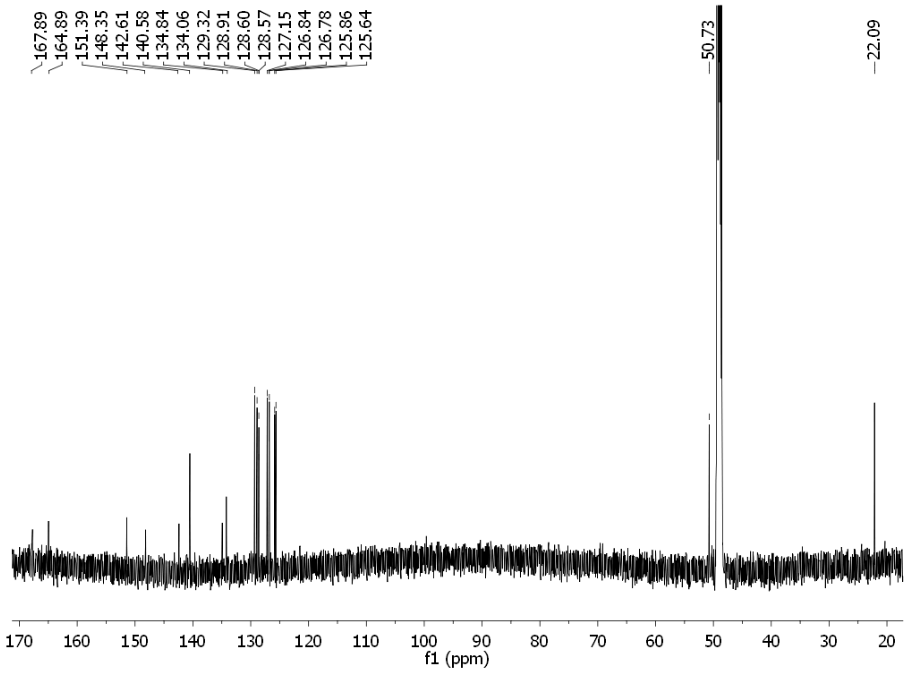

Figure S10. ¹H NMR spectrum of Eu(**1(*R*)**)$_3$ (600 MHz, CD_3CN): (A) full spectrum and (B) zoomed spectrum.

(A)

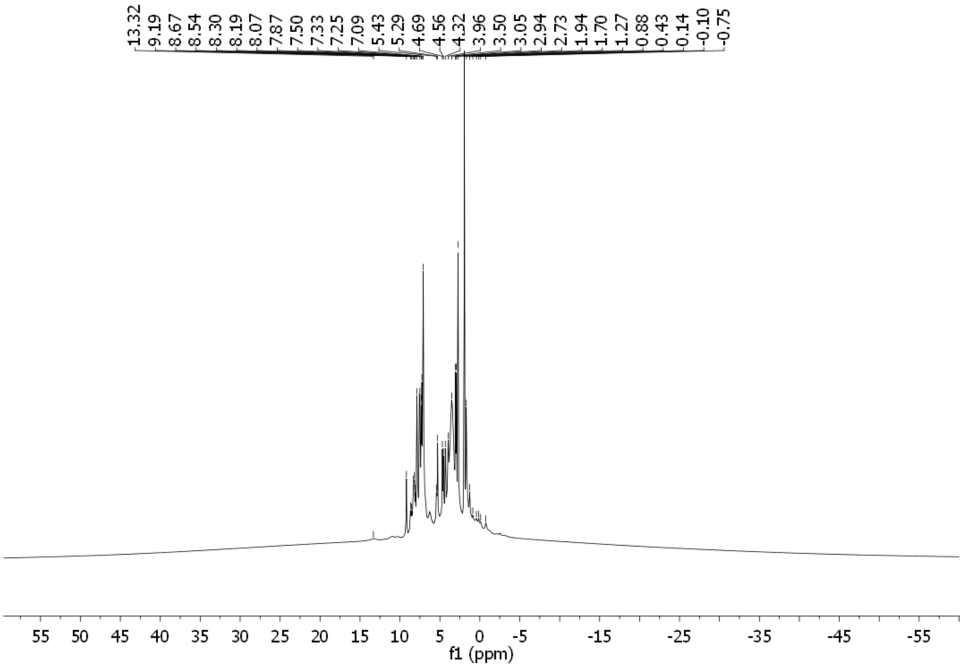

(B)

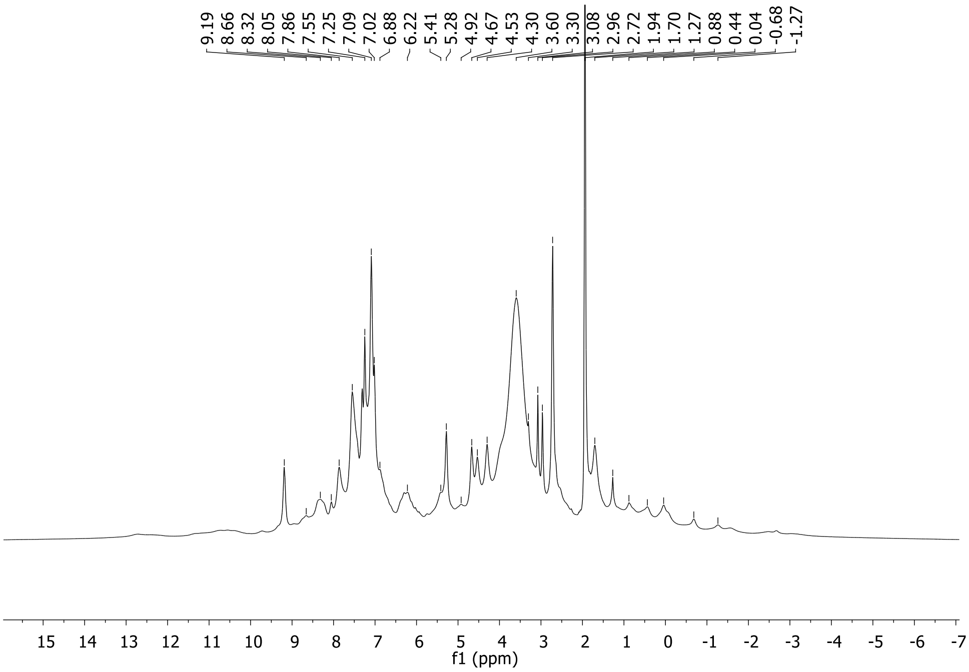

Figure S11. ^{1}H NMR spectrum of Eu(**1(*S*)**)$_3$ (600 MHz, CD_3CN).

(A)

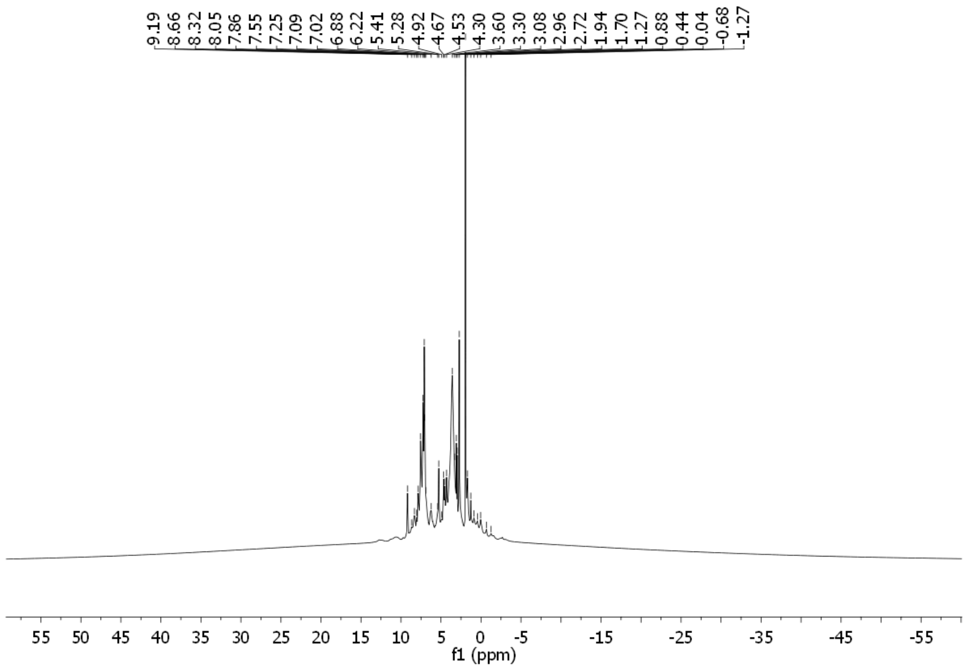

(B)

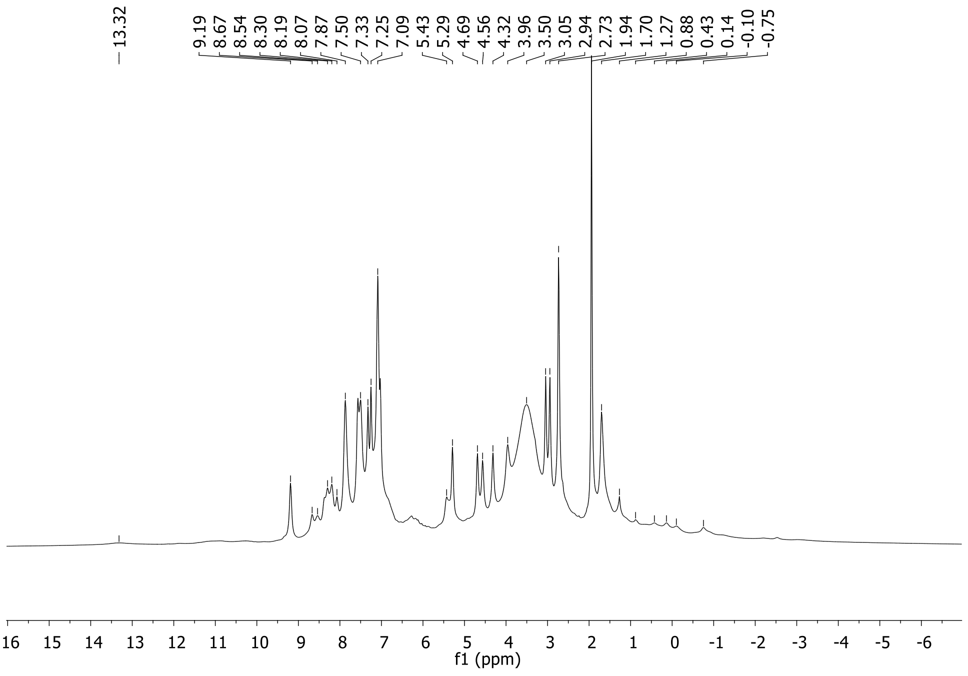

Figure S12. The calculated and experimental isotopic distribution patterns (MALDI-MS$^+$) for (A) **Eu(1(*S*))$_3$** and (B) **Eu(1(*R*))$_3$**.

(A) **(B)**

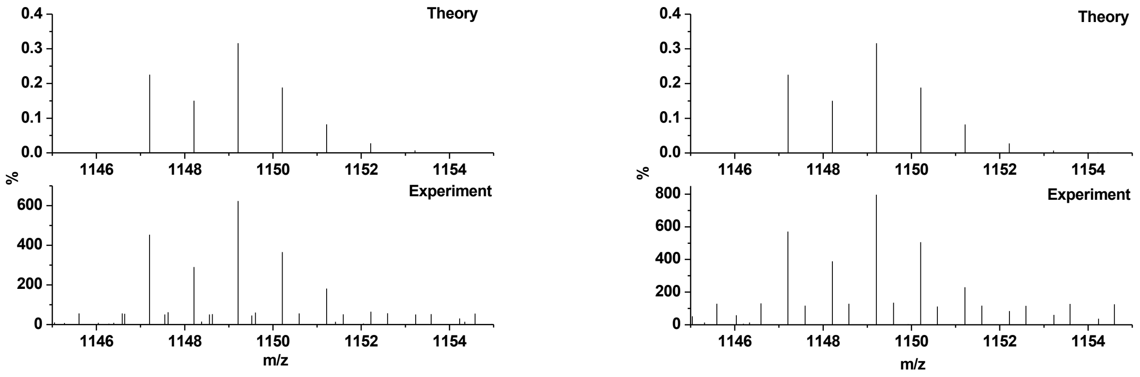

Figure S13. The changes in the absorption spectrum of (A) **1(*S*)** and (C) **1(*R*)** in CH_3CN together with the corresponding linear fit of the changes at 270 nm (B) and (C) upon varying the concentration from 2.5×10^{-5} M to 2.59×10^{-6} M.

(A) **(B)**

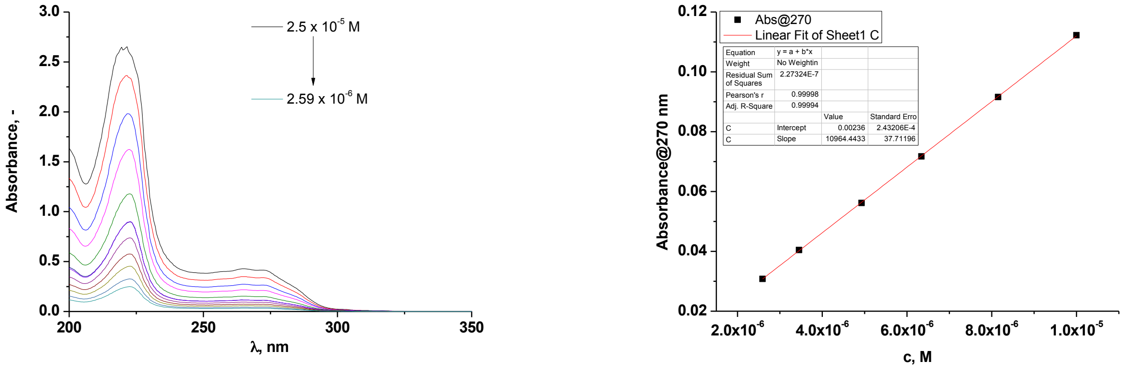

(C) **(D)**

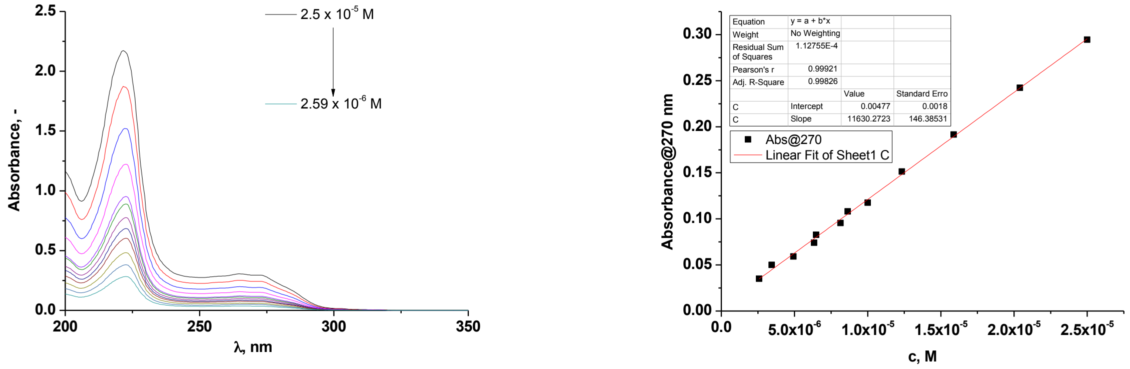

Figure S14. The absorption and fluorescence emission spectra of (A) **1(*S*)** (**–**) and **Eu(1(*S*))$_3$** (**–**) in CH_3CN (c(**1(*S*)**) = 1×10^{-5} M); (B) **1(*R*)** (**–**) and **Eu(1(*R*))$_3$** (**–**) in CH_3CN (c(**1(*R*)**) = 1×10^{-5} M).

(A)

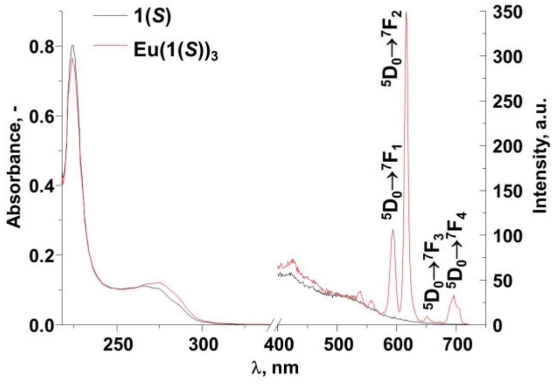

(B)

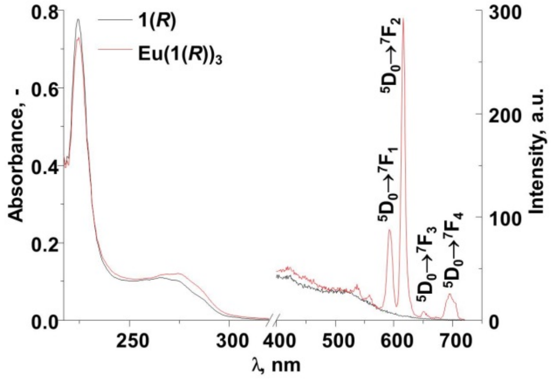

Figure S15. The absorption, excitation and fluorescence emission spectra of **Eu(1(*S*))$_3$** ((A), (B)) and **Eu(1(*R*))$_3$** ((C), (D)) in CH_3CN (c = 2.72×10^{-5} M) and CH_3OH (c = 5.79×10^{-5} M).

(A)

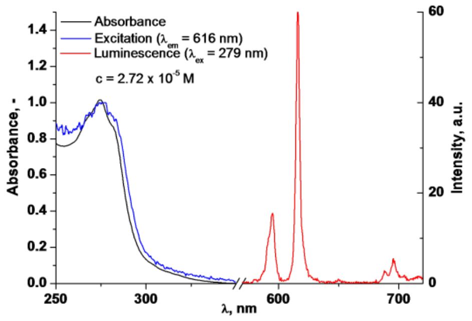

(B)

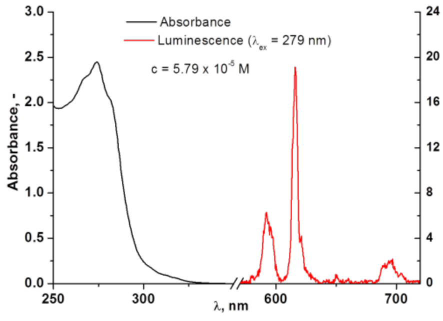

(C)

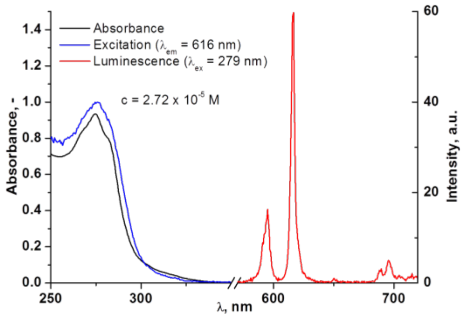

(D)

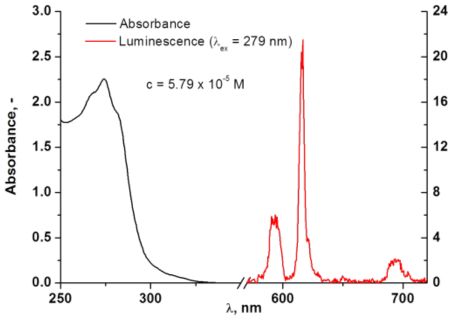

Figure S16. The changes in the (A) absorption, (B) fluorescence and (C) Eu(III)-centred emission spectra of **1(*R*)** (c = 1×10^{-5} M) upon addition of $Eu(CF_3SO_3)_3$ in CH_3CN (25 °C, 0.05 M $(C_2H_5)_4NCl$).

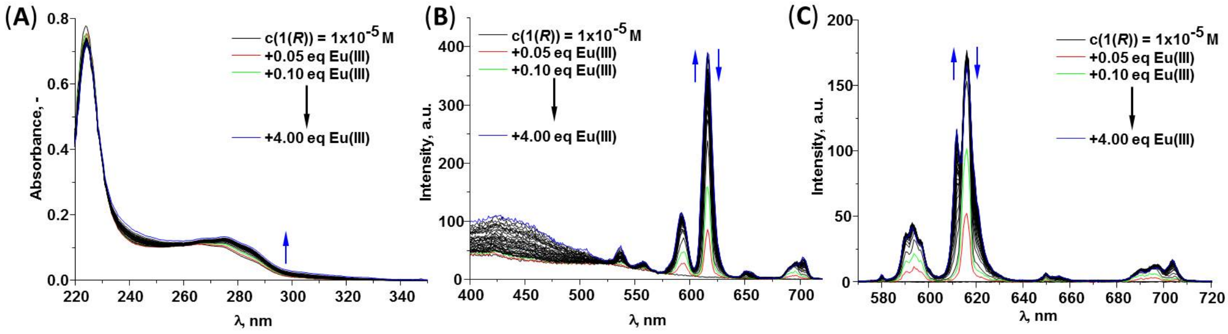

Figure S17. Experimental binding isotherms of the changes in the Eu(III)-centred emission spectrum of (A) **1(*S*)** and (B) **1(*R*)** (c = 1×10^{-5} M) upon addition of $Eu(CF_3SO_3)_3$ in CH_3CN (25 °C, 0.05 M $(C_2H_5)_4NCl$).

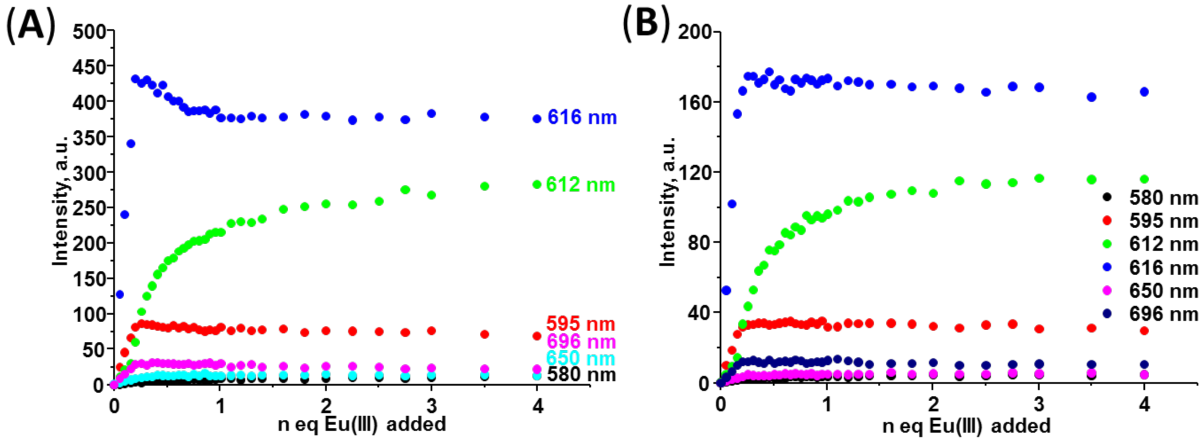

Figure S18. (A) Recalculated emission spectra, (B) experimental binding isotherms (•••) and their corresponding fit (–) by means of SPECFIT for the changes in the fluorescence spectra of **1(*S*)** (c = 1×10^{-5} M) upon addition of $Eu(CF_3SO_3)_3$ in CH_3CN (25 °C, 0.05 M $(C_2H_5)_4NCl$).

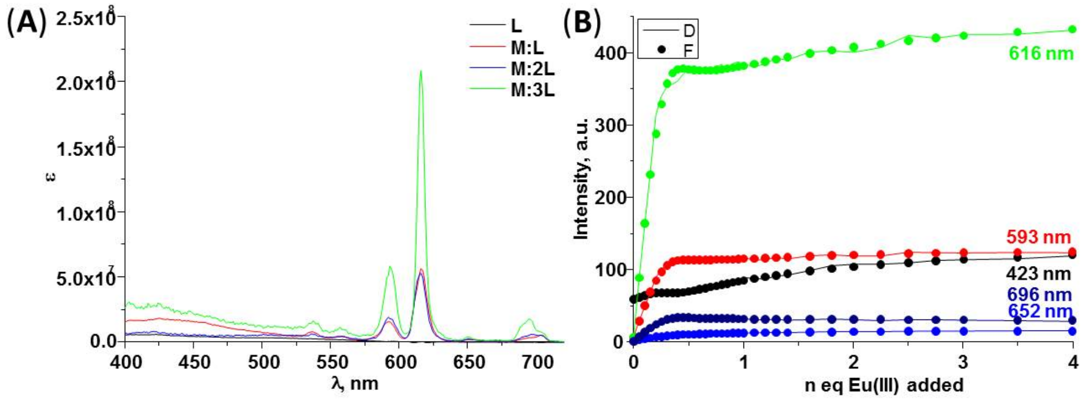

Figure S19. (A) Experimental binding isotherms and their corresponding fit obtained using non-linear regression analysis program SPECFIT, (B) speciation-distribution diagram obtained from the fit of the changes in the absorption spectrum of **1(*R*)** upon addition of $Eu(CF_3SO_3)_3$ in CH_3CN (25 °C, 0.05 M $(C_2H_5)_4NCl$).

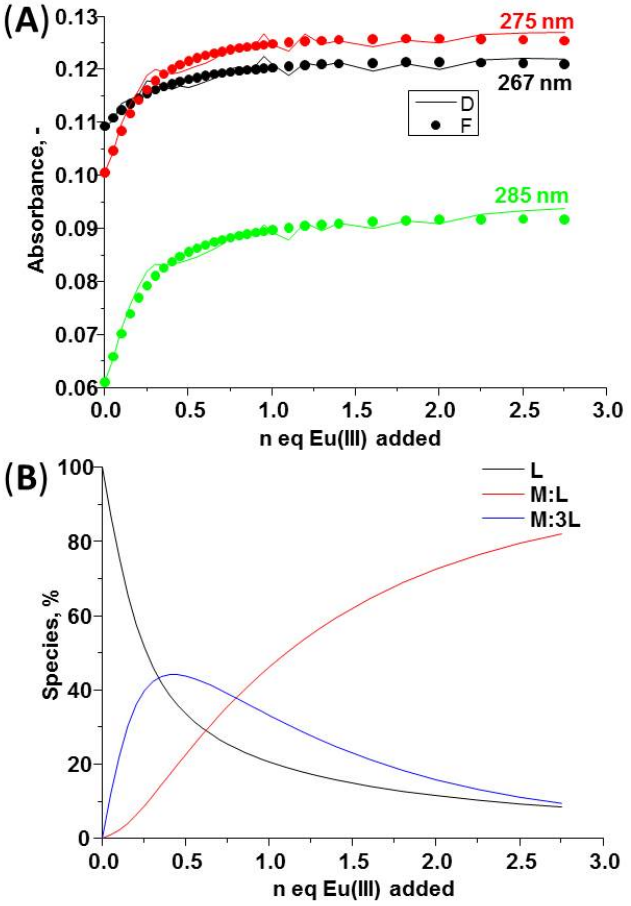

Figure S20. (A) Recalculated emission spectra, (B) experimental binding isotherms (•••) and their corresponding fit (–) by means of SPECFIT for the changes in the fluorescence spectra of **1(*R*)** ($c = 1\times10^{-5}$ M) upon addition of $Eu(CF_3SO_3)_3$ in CH_3CN (25 °C, 0.05 M $(C_2H_5)_4NCl$).

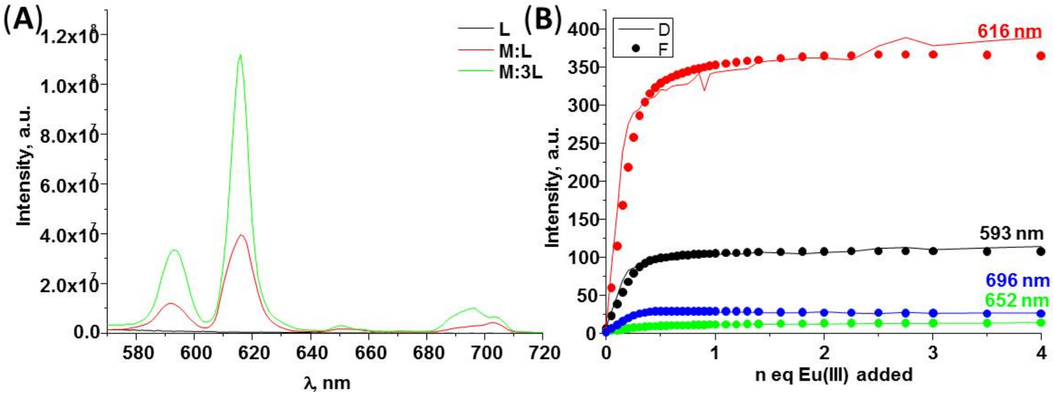

Table S1. Intermolecular hydrogen bond distances (Å) and angles (°) for **1(*R*)** and **1(*S*)**.

D-H…A	Donor-Acceptor		Angle	
	**1(*R*)**	**1(*S*)**	**1(*R*)**	**1(*S*)**
O2-H2···O11$^{\#2}$	2.5709(17)	2.5740(14)	160	159
C7-H7A···O1$^{\#1}$	3.467(2)	3.4744(17)	174	174
C14-H14A···O26	3.249(2) $^{\#3}$	3.2426(17) $^{\#1}$	129	129
O26-H26···O35$^{\#2}$	2.5889(16)	2.5915(14)	156	156
C31-H31A···O25$^{\#1}$	3.479(2)	3.4876(17)	172	172
Symmetry Transformations used to generate equivalent atoms	#1 = x,y,z+1<br>#2 = x,y,z-1<br>#3 x+1,y,z+1			

Table S2. Intermolecular hydrogen bond distances (Å) and angles (°) for **Eu(1(*R*))$_3$**.

D-H…A	Donor-Acceptor	Angle
N2—H2···O16$^{\#1}$	3.031(14)	172.0
N4—H4A···O23	2.88(3)	155.1
N6—H6···O10$^{\#2}$	3.140(12)	152.0
N8—H8···O20	2.78(2)	155.3
N10—H10···O19	2.981(17)	161.0
N12—H12···O1$^{\#2}$	2.83(2)	164.8
O19—H19A···O5	2.78(2)	178.2 (13)
O19—H19B···O17$^{\#1}$	2.98(2)	178.6 (13)
O20—H20A···O8$^{\#3}$	2.80(2)	177.4 (14)
O22—H22A···O8$^{\#4}$	4.00(6)	167 (4)
O23—H23A···O2$^{\#5}$	2.64(3)	178 (2)
O23—H23B···O24	2.80(3)	177 (2)
O24—H24A···O13^{vi}	2.93(2)	125.6 (15)
O24—H24B···O14^{vi}	2.87(3)	111.3 (15)
Symmetry codes: (i) *x*−1/2, −*y*+1/2, −*z*+1; (ii) −*x*+1, *y*, −*z*+3/2; (iii) *x*+1/2, −*y*+1/2, −*z*+1; (iv) −*x*+1/2, −*y*+1/2, *z*−1/2; (v) −*x*+1/2, *y*+1/2, −*z*+3/2; (vi) *x*−1/2, *y*+1/2, *z*.		

Table S3. Selected coordination bond distances (Å) and angles (°) for **Eu(1(*R*))$_3$**.

Eu1—O1	2.389 (14)	O10—Eu2	2.336 (14)
Eu1—O3	2.432 (12)	O12—Eu2	2.468 (14)
Eu1—O4	2.294 (15)	O13—Eu2	2.421 (14)
Eu1—O6	2.415 (15)	O15—Eu2	2.392 (14)
Eu1—O7	2.423 (6)	O16—Eu2	2.352 (15)
Eu1—O9	2.469 (12)	O18—Eu2	2.433 (11)
Eu1—N1	2.542 (15)	N7—Eu2	2.581 (15)
Eu1—N3	2.564 (18)	N9—Eu2	2.558 (19)
Eu1—N5	2.596 (17)	N11—Eu2	2.513 (17)

O4—Eu1—N1	68.1 (5)	O10—Eu2—N11	73.3 (5)
O1—Eu1—N1	65.4 (5)	O16—Eu2—N11	63.1 (5)
O6—Eu1—N1	141.3 (6)	O15—Eu2—N11	138.8 (5)
O7—Eu1—N1	74.6 (4)	O13—Eu2—N11	71.6 (5)
O3—Eu1—N1	63.7 (5)	O18—Eu2—N11	63.5 (5)
O9—Eu1—N1	139.0 (5)	O12—Eu2—N11	136.9 (5)

O4—Eu1—N3	64.2 (7)	O10—Eu2—N9	135.0 (6)
O1—Eu1—N3	137.3 (6)	O16—Eu2—N9	136.4 (6)
O6—Eu1—N3	63.3 (6)	O15—Eu2—N9	62.5 (6)
O7—Eu1—N3	138.3 (5)	O13—Eu2—N9	64.0 (7)
O3—Eu1—N3	72.1 (5)	O18—Eu2—N9	75.6 (5)
O9—Eu1—N3	73.3 (4)	O12—Eu2—N9	74.1 (5)
N1—Eu1—N3	117.6 (6)	N11—Eu2—N9	123.4 (6)
O4—Eu1—N5	141.5 (6)	O10—Eu2—N7	63.6 (5)
O1—Eu1—N5	72.6 (5)	O16—Eu2—N7	77.0 (5)
O6—Eu1—N5	70.8 (5)	O15—Eu2—N7	68.5 (5)
O7—Eu1—N5	62.2 (5)	O13—Eu2—N7	144.8 (5)
O3—Eu1—N5	136.0 (7)	O18—Eu2—N7	135.0 (5)
O9—Eu1—N5	63.2 (6)	O12—Eu2—N7	61.4 (5)
N1—Eu1—N5	121.6 (6)	N11—Eu2—N7	121.0 (5)
N3—Eu1—N5	120.9 (6)	N9—Eu2—N7	115.6 (6)

Figure S21. (A) Changes in CD spectra of **1(*R*)** (–) (c = 1×10^{-5} M, 25 °C) upon addition of $Eu(CF_3SO_3)_3$ and (B) experimental binding isotherms representing the changes in the CD bands of **1(*R*)** (closed circles) and **1(*S*)** (open circles) upon gradual $Eu(CF_3SO_3)_3$ addition in CH_3CN.

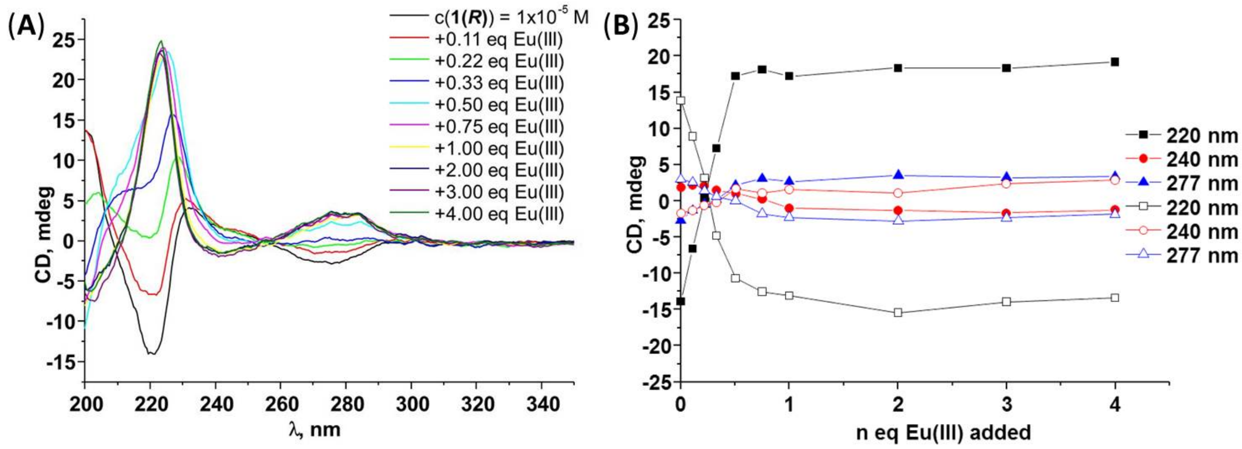

Figure S22. Changes in CD spectra of **1(*S*)** (–) (c = 1×10^{-5} M, 25 °C) upon addition of $Eu(CF_3SO_3)_3$ in CH_3CN.

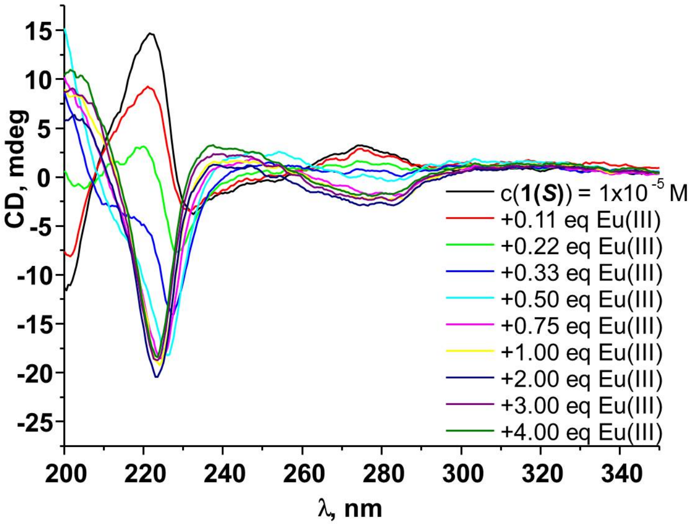

Figure 23. (A) Changes in CD spectra of **1(*R*)** (–) (c = 1×10^{-5} M, 25 °C) upon addition of $Eu(CF_3SO_3)_3$ and (B) experimental binding isotherms representing the changes in the CD bands upon gradual $Eu(CF_3SO_3)_3$ addition in CH_3CN ($(C_2H_5)_4NCl$, 0.05 M).

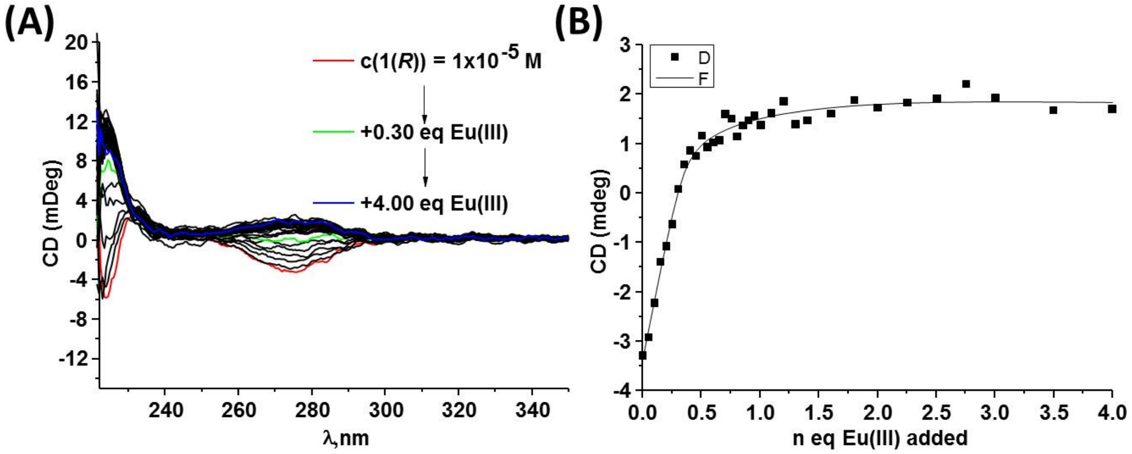

Table S4. Summary of CPL results for $\mathbf{Eu(1(\mathit{S}))_3}$ and $\mathbf{Eu(1(\mathit{R}))_3}$ in methanol at 25 °C.

Electronic transition	Wavelength/nm	g_{lum}	
		**Eu(1(*S*))$_3$**	**Eu(1(*R*))$_3$**
$^5D_0 \rightarrow {}^7F_1$	589	0.16	-0.15
$^5D_0 \rightarrow {}^7F_1$	592	0.19	-0.17
$^5D_0 \rightarrow {}^7F_2$	614	-0.09	0.10
$^5D_0 \rightarrow {}^7F_3{}^*$	650	0.25	-0.14
$^5D_0 \rightarrow {}^7F_4$	693	-0.05	0.05
$^5D_0 \rightarrow {}^7F_4{}^*$	703	0.24	-0.17

* – relatively significant difference for the g_{lum} values between two enantiomers in the case of $^5D_0 \rightarrow {}^7F_3$ and $^5D_0 \rightarrow {}^7F_4$ transitions is most likely due to the noise as both of these are possessing very low in intensity

Figure S24. ^{1}H NMR spectrum of **1(*S*)** (c = 4.26×10^{-4} M; CD_3CN, 600 MHz).

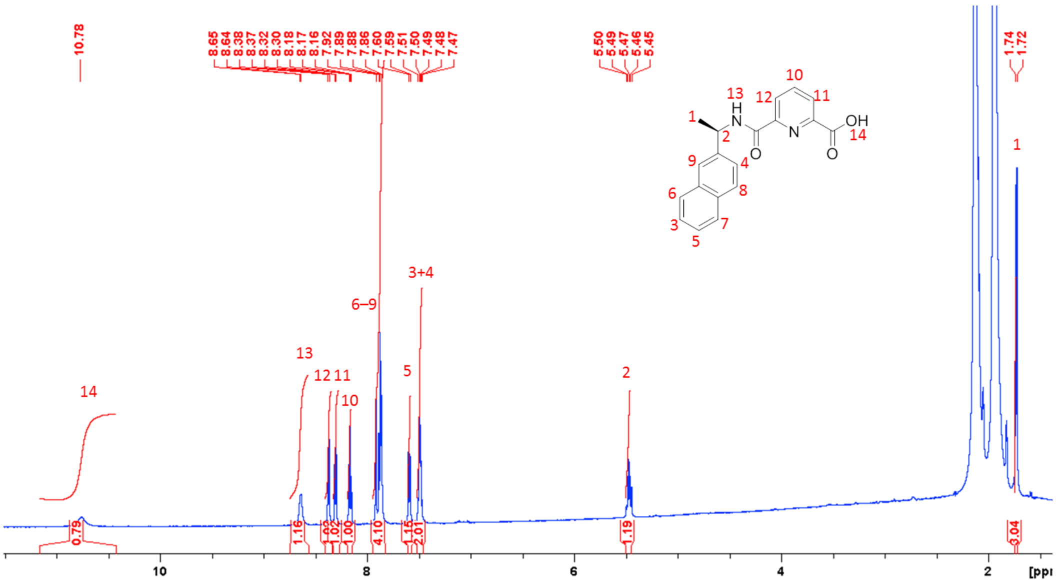

Figure S25. ^{13}C NMR spectrum of **1(*S*)** (c = 4.26×10^{-4} M; CD_3CN, 600 MHz).

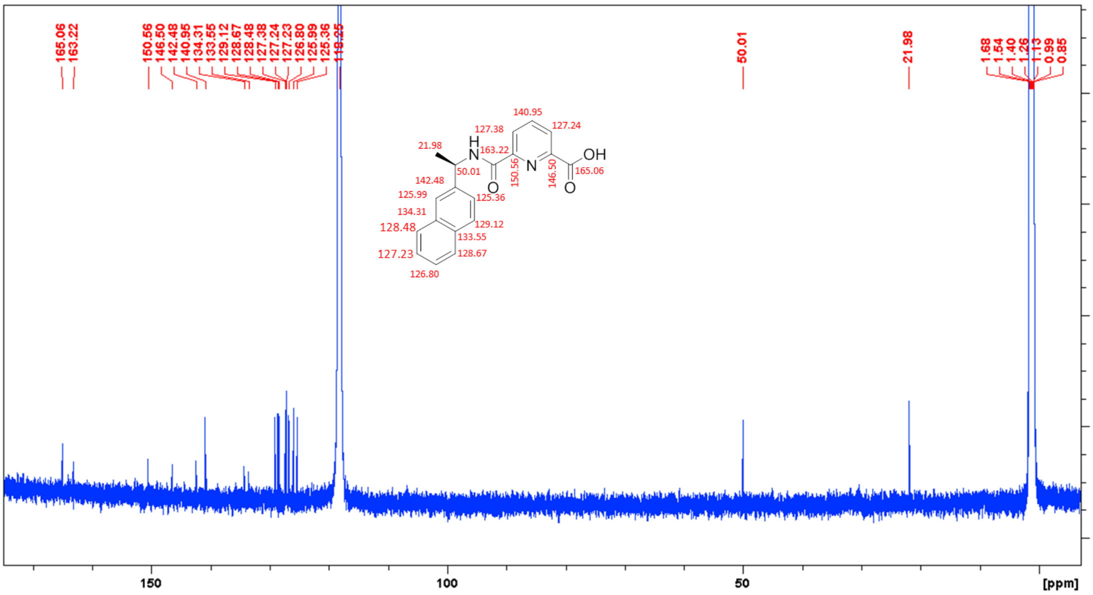

Figure S26. 2D TOCSY spin network for **1(*S*)** (c = 4.26×10^{-4} M; CD_3CN, 600 MHz).

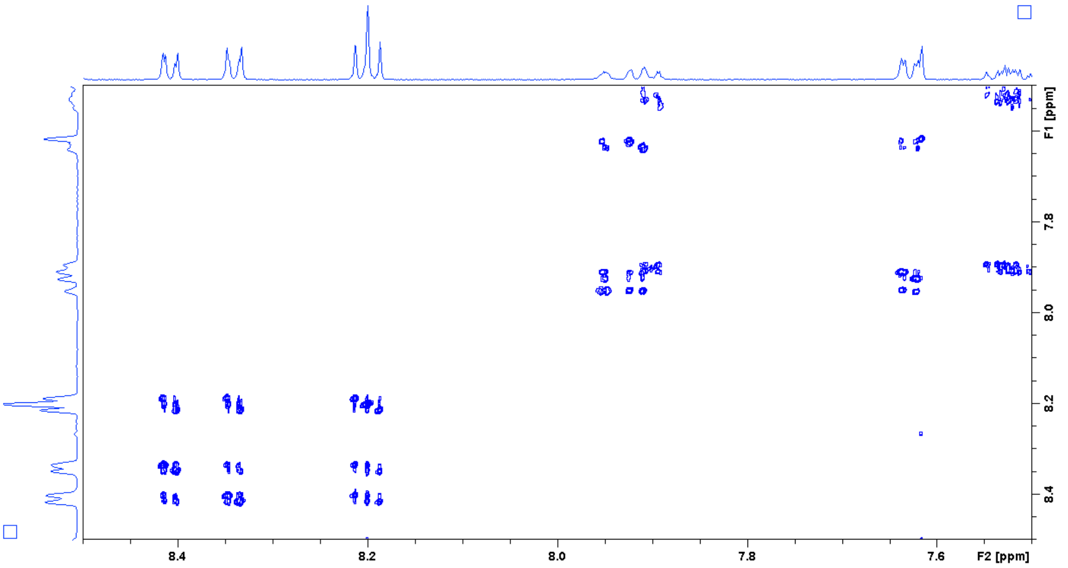

Figure S27. The changes in the ^{1}H NMR spectra of **1(*R*)** (c = 4.26×10^{-4} M) upon gradual addition of $La(CF_3SO_3)_3$ in CD_3CN.

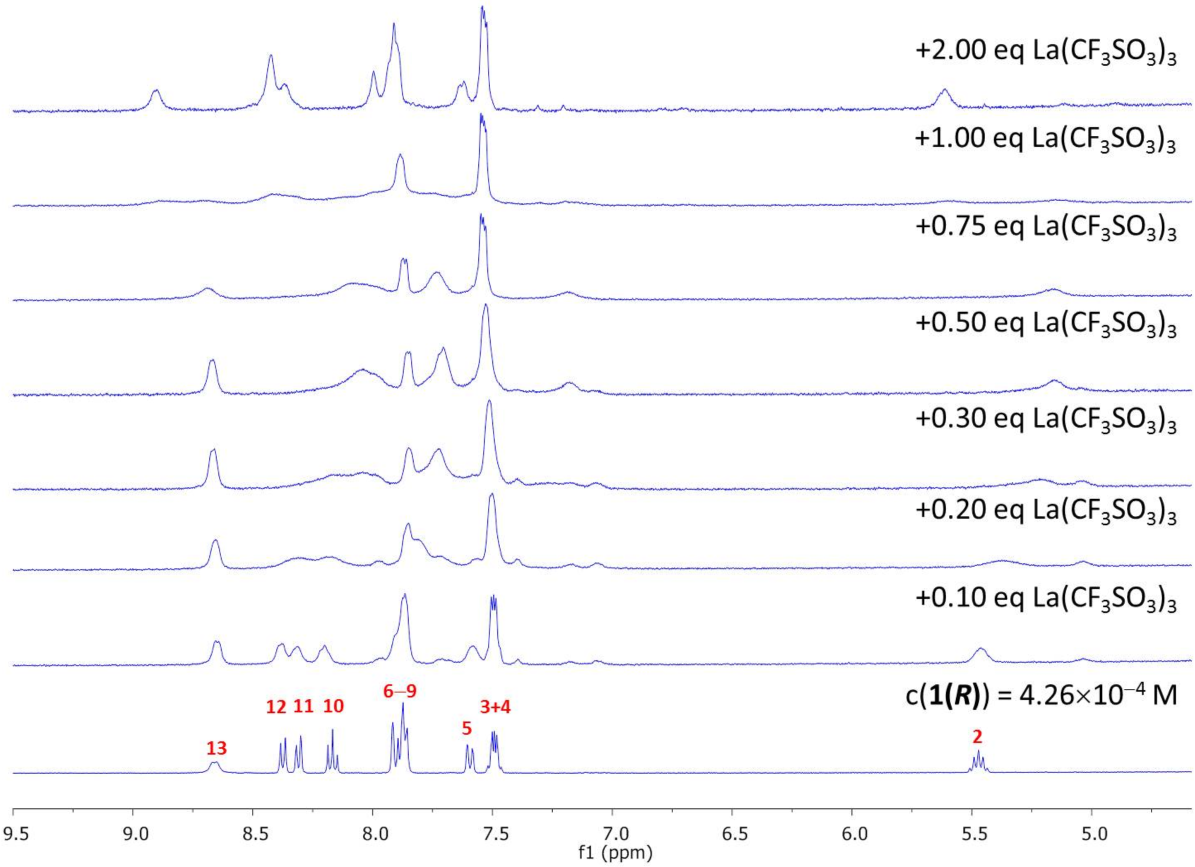

Figure S28. CD spectra of **EuL_3** (**L** = **1(*S*)**, **1(*R*)**; c = 1×10^{-5} M) recorded in CH_3CN at various temperatures: (A) and (C) represent full spectrum range while (B) and (D) zoomed areas.

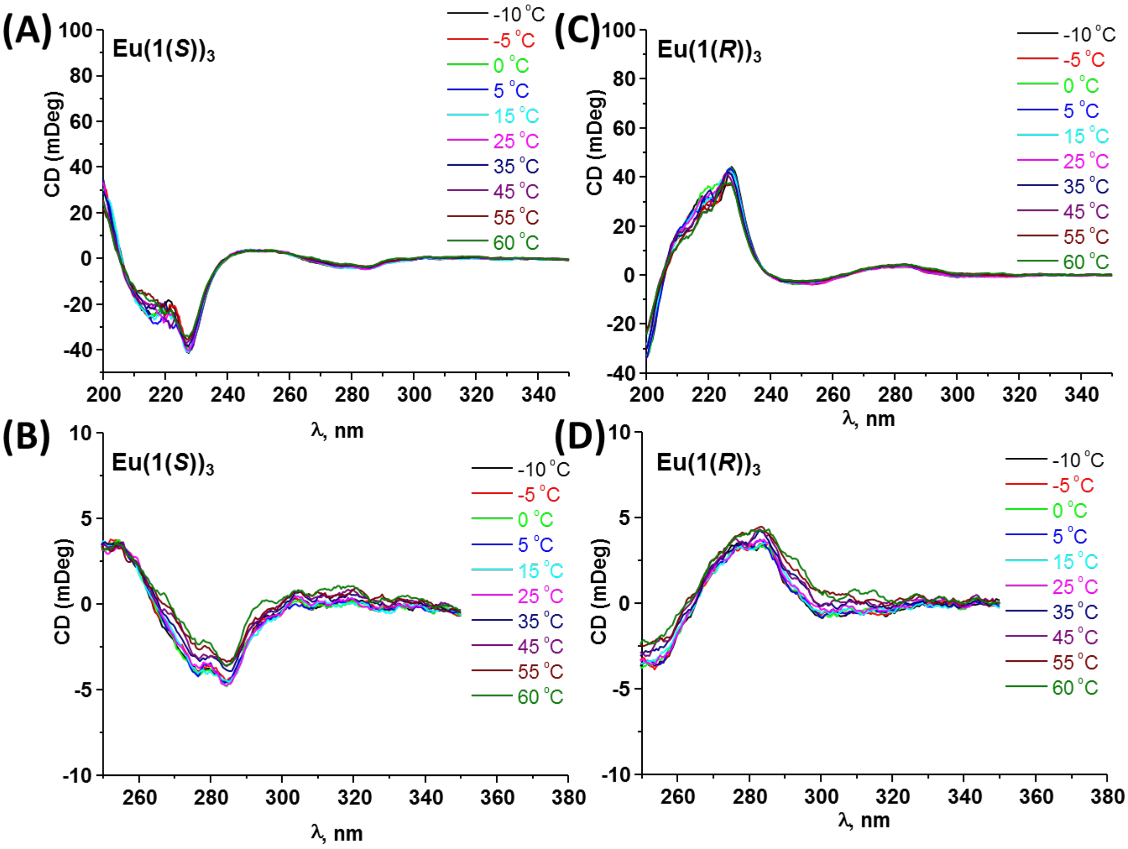

Figure S29. The changes in the main CD bands of EuL_3 (**L** = **1(*S*)**, **1(*R*)**; c = 1×10^{-5} M) in CH_3CN versus temperature of the solution.

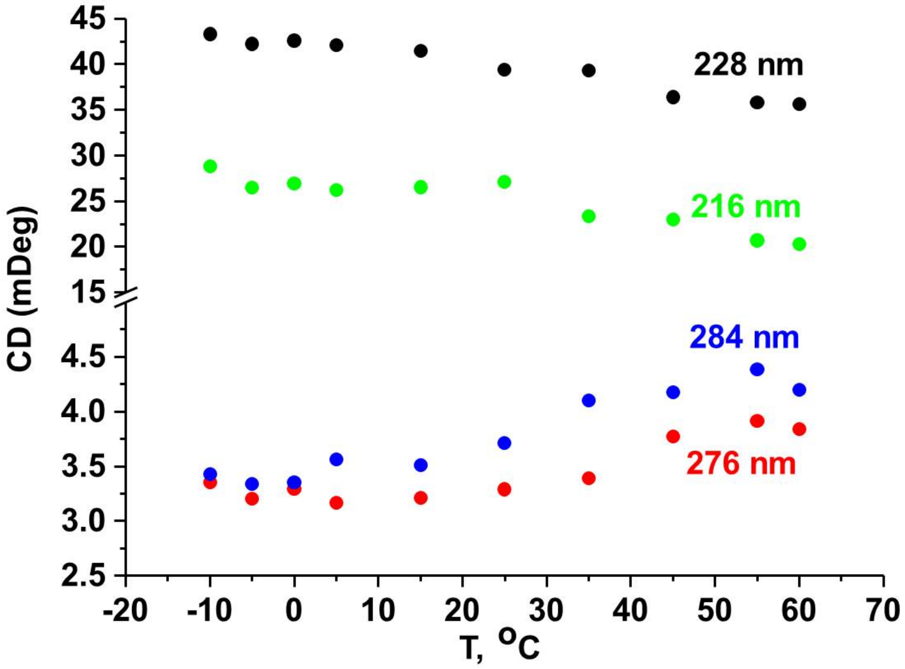

Figure S30. Space filling representation of **Eu(1(R))$_3$** complex. Only fragment 1 is shown for clarity.

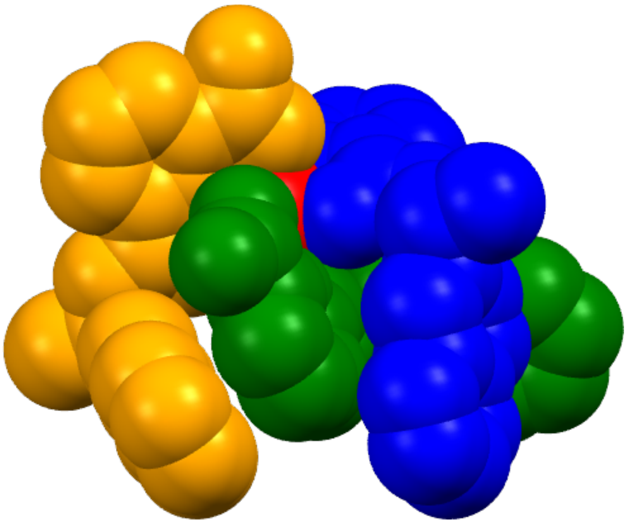

Figure S31. Intramolecular π-π interactions between pyridine and 2-naphthyl moieties within **Eu(1(*R*))$_3$**.

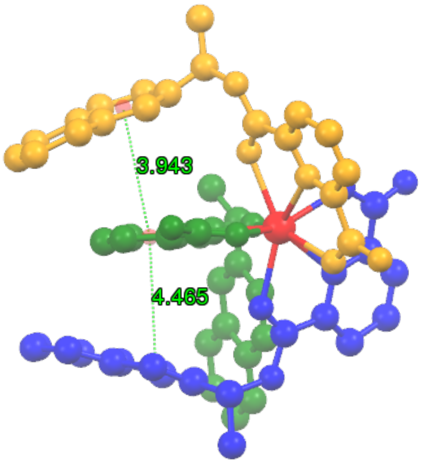

Supplement: Supplementary file 1 [file SC-006-C4SC02474E-s001.pdf]
